# Advances in Yeast Glucan Particles for Oral Drug Delivery

**DOI:** 10.3390/polym18080994

**Published:** 2026-04-19

**Authors:** Hongyi Yin, Yue Wen, Wanneng Li, Shuting Zou, Huanhuan Sun, Tong Chen, Keke Ma, Dean Tian, Jun Liu, Sidan Tian, Mei Liu, Jia Yao

**Affiliations:** 1 Third Hospital of Shanxi Medical University, Shanxi Bethune Hospital, Shanxi Academy of Medical Sciences, Tongji Shanxi Hospital, Taiyuan 030032, China; yinhongyi@sxmu.edu.cn (H.Y.); zoushuting@sxmu.edu.cn (S.Z.); zenmehaobanzenmeban@sxmu.edu.cn (H.S.); datian@tjh.tjmu.edu.cn (D.T.);; 2 Department of Gastroenterology, Tongji Hospital, Tongji Medical College, Huazhong University of Science and Technology, Wuhan 430022, China; wenyue@tjh.tjmu.edu.cn (Y.W.); d202582809@hust.edu.cn (W.L.); 3 National Engineering Research Center for Nanomedicine, College of Life Science and Technology, Huazhong University of Science and Technology, Wuhan 430074, China; chentong@hust.edu.cn (T.C.); m202372780@hust.edu.cn (K.M.); sdtian1616@hust.edu.cn (S.T.); 4 Key Laboratory of Molecular Biophysics of Minister of Education, College of Life Science and Technology, Huazhong University of Science and Technology, Wuhan 430074, China

**Keywords:** composite delivery systems, yeast glucan particles system, polymer modification, macrophage targeting, immunoregulation

## Abstract

In recent years, yeast glucan particles (YGPs) have garnered significant attention as novel oral drug delivery carriers, owing to their superior biocompatibility, specific targeting capabilities, and intrinsic immunomodulatory properties. The yeast cell wall is primarily composed of β-glucan and mannan, with minor amounts of proteins and lipids. Among these, β-1,3-glucan serves as the pivotal functional component. It not only provides a physical barrier protecting payloads from gastric acidity and enzymatic degradation but also functions as a targeting ligand. By specifically binding to M cells in Peyer’s patches and Dectin-1 receptors on macrophages and dendritic cells, β-1,3-glucan facilitates precise drug delivery to gut-associated lymphoid tissue (GALT) or macrophage-rich inflammatory sites. Consequently, β-1,3-glucan-based YGPs demonstrate immense potential in oral targeted delivery systems for macrophage-associated pathologies. However, native YGPs, constrained by their inherent porous architecture and relatively simple physicochemical properties, often fall short of meeting the complex requirements for precise encapsulation, controlled release, and multifunctionality. To address these limitations, current research is actively exploring the functionalization of YGPs with various composite materials to engineer advanced delivery platforms. This review introduces the composition, structural characteristics, and fabrication methodologies of YGPs, alongside their specific merits and limitations in oral drug delivery. Furthermore, it critically analyzes strategies for modifying YGPs with composite materials to overcome delivery barriers. Finally, the review discusses their therapeutic applications across various diseases and outlines future developmental trends.

## 1. Introduction

Oral administration, the most prevalent route of drug delivery, has long been the preferred choice for pharmaceutical development and clinical application due to its non-invasive nature, high patient compliance, and manufacturing cost-effectiveness [1]. Efficient oral drug delivery systems can significantly improve patients’ quality of life, ensure better adherence to treatment regimens, and alleviate the burden on healthcare systems. This is particularly critical for the long-term management of chronic conditions such as inflammatory bowel disease (IBD), diabetes, hypertension, autoimmune disorders, and certain malignancies.

Therefore, the development of highly efficient, safe, and targeted oral delivery systems is pivotal for enhancing global health outcomes and addressing escalating therapeutic needs. Driven by tremendous advances in biotechnology and materials science, oral DDS are evolving from conventional dosage forms (e.g., tablets and capsules) to intelligent delivery platforms, aiming for targeted distribution, precise release kinetics, enhanced bioavailability, and minimized systemic toxicity [2].

Despite these numerous advantages, the successful delivery of oral drugs faces a series of formidable challenges, primarily stemming from the complex physiological environment of the gastrointestinal (GI) tract and the physicochemical properties of the drugs themselves [1,2].

First, the harsh conditions within the GI tract constitute the primary obstacle. The highly acidic gastric environment (pH 1–3) and the presence of pepsin pose a severe threat to acid-labile or enzyme-sensitive drugs, such as proteins, peptides, nucleic acids, and certain small molecules. These agents undergo substantial inactivation before reaching their absorption sites [1]. For instance, biological macromolecules like insulin are inherently susceptible to degradation in the stomach, resulting in negligible oral bioavailability [3]. Furthermore, although the pH rises as drugs enter the small intestine, they encounter further challenges from pancreatic enzymes, bile salts, and the intestinal mucosal barrier.

Second, low bioavailability represents another core issue. According to the Biopharmaceutics Classification System (BCS), efficient oral absorption is dictated not only by drug aqueous solubility but also by intestinal permeability [4]. Many drugs exhibit poor oral bioavailability because they fail to cross the complex intestinal mucosal barrier. Specifically, the dense intestinal epithelial cell monolayer and highly selective tight junctions severely restrict the paracellular and transcellular passage of hydrophilic, highly polar, or large-molecular-weight therapeutics (e.g., proteins and peptides) [5]. Furthermore, the thick viscoelastic mucus layer acts as a dynamic steric and diffusional barrier, trapping drug particles before they can even reach the underlying epithelium [6]. Coupled with significant hepatic first-pass metabolism and rapid clearance by localized efflux transporters, drugs are often prematurely inactivated or expelled, drastically reducing their effective systemic concentration. These multifaceted challenges have prompted researchers to actively investigate advanced microencapsulation technologies and biomimetic carriers to simultaneously enhance drug solubility, facilitate mucosal penetration, and ultimately boost oral bioavailability [7,8,9,10].

Furthermore, non-specific distribution remains a critical bottleneck. Most oral drugs are absorbed non-selectively, distributing to healthy tissues, which may induce systemic side effects and reduce the effective concentration at the pathological site. For conditions requiring localized action, such as IBD or colon cancer, lack of targeting significantly compromises therapeutic efficacy and exacerbates adverse reactions [11,12,13,14,15,16,17]. Additionally, rapid clearance limits the duration of action, necessitating frequent dosing, which negatively impacts patient compliance.

These multifaceted challenges collectively constitute the major bottlenecks in the research and development of oral drug delivery systems, driving researchers to continuously explore innovative strategies and novel vehicles to overcome these difficulties.

**Figure 1:**
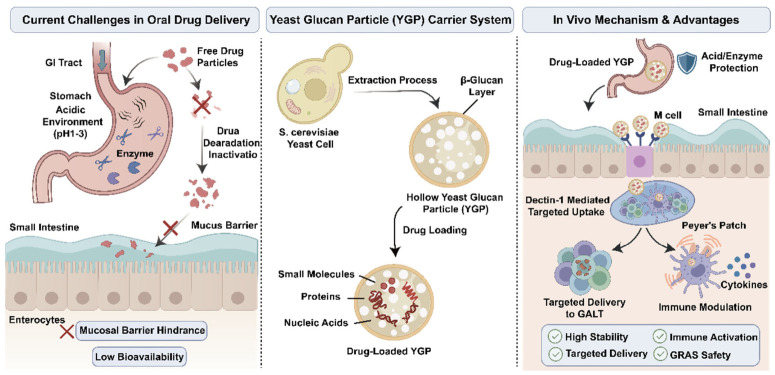
The Yeast Glucan Particle (YGP) system for targeted oral drug delivery. Comparison of free drug limitations versus the YGP carrier strategy. While free drugs suffer from gastric degradation and mucosal barrier hindrance (**left**), YGPs derived from extracted S. cerevisiae cells provide a protective, hollow β-glucan shell for encapsulating small molecules and biologics (**middle**). The YGP system protects the payload from gastric acidity and exploits Dectin-1-mediated uptake by M cells to target GALT, ensuring high stability, targeted delivery, and immune activation (**right**).

In the face of the numerous challenges confronting oral drug delivery systems, YGPs as a novel bio-derived delivery vehicle, have received widespread attention from the academic community in recent years [18,19,20,21]. Their distinctive structural and biological characteristics provide significant potential as delivery vehicles for overcoming the harsh gastrointestinal environment, facilitating targeted distribution, and promoting immunomodulation (Figure 1).

The concept of YGPs stems from the purification of the Saccharomyces cerevisiae cell wall. Through specific extraction processes that remove cytoplasmic components, hollow and porous microspheres primarily composed of β-glucan are obtained [18,19]. This natural hollow architecture serves as an ideal reservoir for drug encapsulation, effectively shielding therapeutics from gastric acid and digestive enzymes. Beyond physical protection, YGPs possess a dual functionality: they act not only as a protective barrier but also as an active targeting vector. β-glucan is specifically recognized and internalized by M cells in Peyer’s patches and by Dectin-1 receptors on macrophages and dendritic cells [18,22,23,24]. This specific recognition mechanism endows YGPs with intrinsic targeting capabilities, enabling precise delivery to GALT or macrophage-rich inflammatory sites [18], thereby optimizing therapeutic outcomes while mitigating systemic side effects [25].

Moreover, β-glucan exhibits significant immunomodulatory activity, capable of activating the innate immune system and inducing cytokine release. It can also serve as a potent vaccine adjuvant [26,27,28,29,30,31]. These characteristics broaden the application prospects of YGPs in cancer immunotherapy and vaccine delivery. Importantly, Saccharomyces cerevisiae is classified as “Generally Recognized As Safe” (GRAS) by the U.S. FDA [24]. Its cell wall components possess excellent biocompatibility, biodegradability, and low toxicity, laying a solid safety foundation for the clinical translation of YGP-based delivery systems [32,33,34,35,36].

In summary, by virtue of their unique structure, natural targeting specificity, immunomodulatory activity, and superior biosafety, YGPs have become a highly attractive novel vehicle in the field of oral drug delivery systems.

## 2. Composition and Structural Characteristics of Yeast Cell Wall

**Figure 2:**
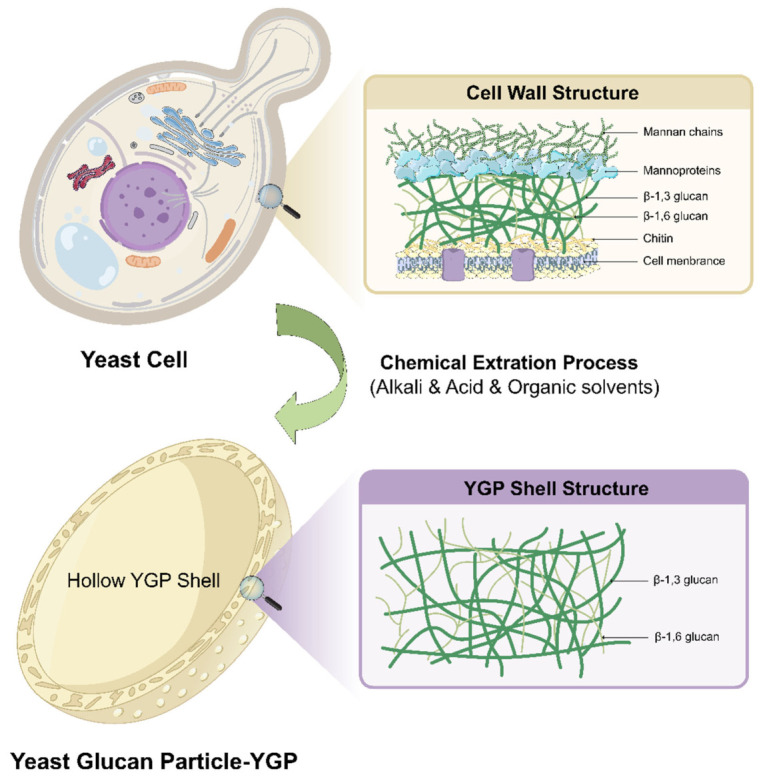
Schematic Diagram of Yeast Cell Wall Structure.

The yeast cell wall is a complex and robust multilayered structure, spanning approximately 25 nm in thickness and accounting for 20–30% of the cell’s dry weight. This structure confers specific morphology to the yeast cell and provides protection against osmotic stress and environmental insults. In terms of chemical composition, the cell wall is predominantly composed of polysaccharides, which constitute approximately 80–90% of the total mass, with the remainder consisting of proteins and minor amounts of lipids [18]. These constituents are covalently cross-linked to form a stratified network architecture (Figure 2).

The innermost layer of the cell wall acts as its structural scaffold and is composed primarily of β-glucan. β-glucan is a high-molecular-weight polysaccharide consisting of glucose units linked by β-glycosidic bonds. In yeast, the structure features a β-1,3-glucan backbone cross-linked with β-1,6-glucan side chains to form a three-dimensional network [19,37]. Kang et al., utilizing modern solid-state NMR (ssNMR) techniques, demonstrated that β-glucans in the fungal cell wall predominantly adopt a triple-helical conformation. These helices extensively cross-link with components such as chitin to construct a highly hydrated, flexible, and viscoelastic three-dimensional matrix [38]. This dynamic framework not only provides the flexibility required to withstand turgor pressure but also serves as the core scaffold for anchoring various surface proteins. Furthermore, this robust reticular architecture confers exceptional physicochemical stability to YGPs, enabling them to maintain structural integrity within the gastrointestinal tract and protect encapsulated bioactive substances. Crucially, β-glucan is the pivotal molecule responsible for the targeting function of the YGPs. It is specifically recognized by Dectin-1, a pattern recognition receptor (PRR) expressed on the surface of mammalian immune cells [24]. This specific recognition serves as the molecular basis for the targeted delivery of YGPs to antigen-presenting cells (APCs), such as macrophages and dendritic cells. Consequently, this mechanism holds significant promise for the treatment of macrophage-associated disorders, including inflammatory bowel disease and cancer.

The outer layer of the cell wall is primarily composed of highly glycosylated mannoproteins. These proteins are covalently linked to the β-glucan network via glycosylphosphatidylinositol (GPI) anchors and β-1,6-glucan, with mannan chains of varying lengths extending outward to form the cell surface [19]. Mannoproteins confer hydrophilicity and a negative charge to the yeast cell wall while also participating in various biological processes such as cell adhesion, flocculation, and immune recognition. In drug delivery process, the presence of mannoproteins may affect the binding efficiency of β-glucan to Dectin-1 receptors. Therefore, during the preparation of YGPs, the outer mannoproteins are typically removed via chemical or enzymatic treatment to expose the inner β-glucan layer, thereby enhancing targeting capability [39].

Additionally, a small amount of chitin exists in the innermost layer of the cell wall; this substance is distributed mainly in specific regions, such as bud scars, and plays a crucial role in maintaining cell wall integrity [19,40].

In summary, the unique structure of the yeast cell wall, particularly its β-glucan backbone, makes it a highly promising carrier for oral drug delivery. It not only provides physical protection for drugs but also enables targeted delivery and immunomodulation through specific recognition mechanisms, laying a solid foundation for the development of novel and efficient oral drug delivery systems.

## 3. Preparation of YGPs

The core of YGPs preparation lies in removing intracellular substances from intact yeast cells while preserving their robust cell wall skeleton, forming a hollow structure for drug encapsulation. This process typically involves a series of chemical and physical treatment steps aimed at maximizing the purification of cell wall components and maintaining their structural integrity. Currently, methods for preparing YGPs or extracting β-glucan from yeast cell walls are primarily categorized into chemical methods, physical ultrasonic-assisted extraction, and enzymatic treatment methods.

### 3.1. Chemical Methods

**Figure 3:**
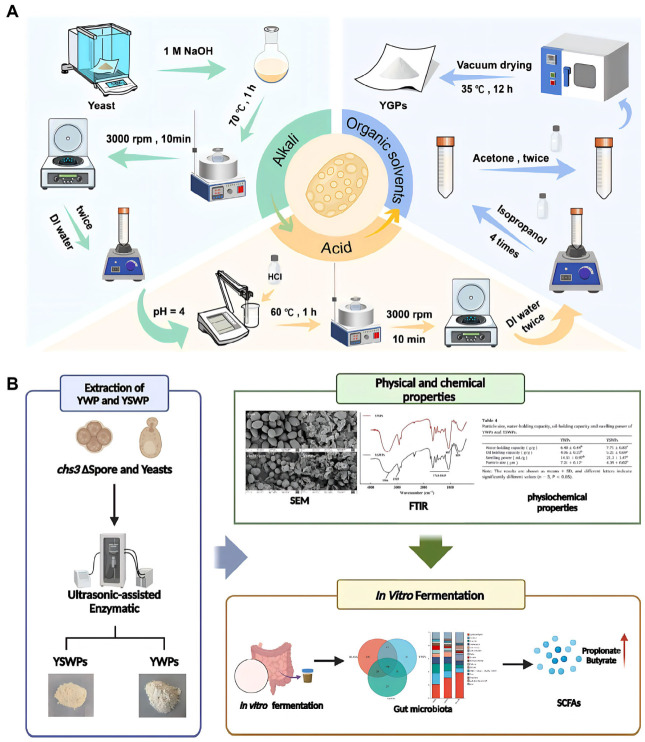
Preparation and characterization of yeast cell wall β-glucans via different extraction methods. (**A**) The traditional chemical extraction process involving alkali–acid–solvent treatments to obtain hollow yeast glucan particles (YGPs). (**B**) Ultrasonic-assisted enzymatic approach for isolating yeast wall particles (YWPs) [44]. Copyright © 2025 The Authors. Published by Elsevier B.V.

The chemical method, typically the “alkali-acid method,” utilizes acid-alkali solutions to remove impurities like mannoproteins, lipids, and glycogen, isolating the insoluble glucan backbone. The process (Figure 3A) involves alkaline treatment (e.g., NaOH) to hydrolyze cytoplasmic proteins and mannoproteins. This is followed by acid treatment (e.g., HCl) to remove acid-soluble impurities, yielding hollow β-glucan and chitin microcapsules. Subsequent washing with organic solvents removes lipids and adjusts pore size [41]. To maximize YGPs’ biological activity, structural integrity, and drug-loading capacity, modern research has focused on fine-tuning specific parameters within this extraction sequence. For instance, Yang et al. optimized the temperatures for the alkali and acid treatments to 70 °C and 60 °C, respectively, each with a duration of 1 h. This optimized procedure not only effectively removed intracellular contents to expand the YGPs’ cavities but also substantially preserved the structural integrity of the cell wall skeleton, yielding a high β-glucan content of 73.32 ± 3.18%. Furthermore, the processed YGPs exhibited an optimal zeta potential of −10.8 mV. The synergistic interaction between this optimized negative surface charge and the enlarged internal cavity significantly enhanced the electrostatic adsorption and internal encapsulation of positively charged drugs, such as berberine, ultimately achieving a remarkably high encapsulation efficiency (EE) of 78.68% and a drug-loading capacity (LC) of 8.34% [39]. Amer et al. optimized the process using 1 M NaOH at 80 °C followed by acetic acid extraction, yielding β-glucan with a high purity of 85%, compared to 80% via water extraction. Furthermore, the extract demonstrated superior antibacterial properties by exhibiting minimum inhibitory concentrations (MICs) of 0.39 and 0.19 mg/mL against *Methicillin-resistant Staphylococcus aureus* (MRSA) and *Pseudomonas aeruginosa* (*P. aeruginosa*), respectively, alongside a positive immunomodulatory effect mediated through the high induction of TNFα, IL-6, IFN-γ, and IL-2 [42]. Following the acid-base extraction, Saloň et al. incorporated sequential washing steps using isopropanol and acetone to further purify the YGPs. However, this process removed the hydrophilic components of the cell wall, resulting in a significant increase in the surface hydrophobicity of the YGPs (with the water contact angle increasing from 37° to 75°), which consequently increased the tendency of the YGPs to agglomerate over prolonged periods. Nevertheless, a brief sonication treatment effectively disrupted these agglomerates, enabling the resulting YGPs to demonstrate excellent suspension stability during the initial 2 h following resuspension, a period during which their volume mean particle size (approximately 7 μm) remained essentially unchanged [43].

In summary, these targeted optimizations demonstrate that, through the precise modulation of the conventional alkali–acid process, it is possible to fabricate structurally robust, high-purity, and functionally stable YGPs, thereby making them an excellent drug delivery carrier.

This method offers low cost and scalability, maximally preserving the hollow structure, though harsh conditions may cause some damage to the β-glucan fine structure.

### 3.2. Physical Ultrasonic-Assisted Extraction

Ultrasound-assisted extraction technology primarily utilizes the acoustic cavitation effect generated when ultrasonic waves propagate through liquid media. When high-intensity ultrasound passes through a yeast suspension, tens of thousands of microscopic bubbles form within the liquid. Under the influence of the acoustic field, these bubbles rapidly grow and violently implode, generating instantaneous micro-regions of extreme heat (>5000 K) and pressure (>100 MPa), accompanied by intense microjets and shear forces [45].

Compared to purely chemical methods, the mechanical shear forces generated by ultrasound more effectively disrupt the dense chitin-glucan network structure of yeast cell walls, increasing solvent permeability. This accelerates the release of intracellular active substances and the dissolution of cell wall polysaccharides. Studies indicate that ultrasonic treatment significantly shortens extraction time and enhances extraction efficiency. For instance, Yuan et al. found that incorporating ultrasonic assistance (18 W/mL, 20 min) during enzymatic extraction of yeast β-glucan tripled its solubility (75.35%), enhancing both reaction rate and enzyme-substrate affinity [46]. Furthermore, ultrasonication’s physical effects can partially reduce polysaccharide molecular weight, improving water solubility and dispersibility—a critical advantage for subsequent drug delivery system development [47].

Despite ultrasonication’s superior cell disruption capabilities, multiple studies indicate that relying solely on ultrasonic treatment often fails to yield high-purity yeast cell wall components. This is because the outer-layer mannoproteins and inner-layer glucans of the yeast cell wall are tightly connected via covalent bonds. Simple physical-mechanical forces struggle to completely sever these chemical bonds, resulting in high levels of protein impurities in the extract. Therefore, current strategies predominantly employ combined “physical-chemical” or “physical-biological” techniques.

### 3.3. Enzymatic Treatment Methods

Compared to traditional acid-base chemical methods and mechanical disruption processes, enzymatic extraction is widely recognized as a gentler and more efficient method for isolating yeast β-glucan. This approach utilizes highly specific enzymes to selectively degrade targeted components within the cell wall, thereby inducing cell wall disintegration and releasing intracellular substances. Ultimately, this process yields high-purity yeast cell wall glucan. After comparing various strategies for extracting β-D-glucan, Varelas et al. noted that the introduction of exogenous proteases is a key step in improving product purity. Although adding an enzymatic hydrolysis step increases the complexity of the extraction process, this method allows for more thorough removal of residual proteins tightly bound to the cell wall. Experimental data show that the purity of β-glucan treated with proteases can consistently reach 93.12% to 94.0%; in contrast, the purity of β-glucan extracted by conventional alkaline methods without exogenous enzyme treatment typically fluctuates around 91.12% [48]. Similarly, To further remove lipids and residual proteins tightly bound to glucan, Borchani et al. employed a combined enzymatic extraction method using proteases and lipases. This approach selectively degrades peptides and fatty acids within yeast cells under mild conditions, yielding higher-purity yeast glucan. Experimental data demonstrated that this sequential treatment increased the β-glucan purity from an initial 13.4% in native cell walls to 53.4% in the final fraction, with the glucose content reaching 79.0% on a dry weight basis. The efficacy of this purification was further evidenced by the significant reduction in proteins (from 27.6% to 8.44%) and mannans (from 9.28% to 2.11%) [49]. To further enhance extraction efficiency and reduce reaction time, recent studies have integrated enzymatic methods with physical assistance. For instance, research by Yan et al. and Ma et al. demonstrated that ultrasound-assisted processing utilizes cavitation effects to disrupt the spatial structure of cell walls, increasing the contact area between enzyme molecules and substrates and thereby boosting enzymatic reaction efficiency. This “physico-biological” coupling technology (Figure 3B) not only improves extraction efficiency but also significantly enhances the water solubility of glucan, making it more suitable for research in drug delivery systems and functional foods [44,47].

Enzymatic processing conditions are mild, better preserving β-glucan’s natural triple-helix structure and preventing chain degradation. Theoretically, this may yield higher biological activity. However, enzymatic preparation incurs higher costs, exhibits relatively low reaction efficiency, and is significantly constrained by enzyme purity and activity, which critically impact final product quality, limiting its widespread application.

Selecting an appropriate extraction strategy is critical for preserving the structural integrity of YGPs. Retaining their unique hollow architecture is essential, as these cavities form the basis for YGPs’ function as delivery carriers. Furthermore, the inherent porosity of extracted YGPs necessitates strategic surface functionalization to minimize premature cargo leakage and optimize loading efficiency.

## 4. Drug-Loading Methods for YGPs

Encapsulation efficiency (EE) and Loading capacity (LC) are key indicators for evaluating the success of drug loading into YGPs. EE is defined as the percentage of drug successfully encapsulated within YGPs relative to the total initial drug dosage, reflecting the utilization efficiency of the administered drug. LC is the percentage of the total drug content that is encapsulated relative to the total mass of the drug-loaded particles, indicating the payload density of the carrier. The EE and LC of drug-loaded YGPs are generally calculated using the following formulas [50]:

$$EE\left(\%\right)=\frac{amount\;of\;drug\;in\;particles}{total\;amount\;of\;drug\;added}\times 100\%$$

$$LC\left(\%\right)=\frac{amount\;of\;drug\;in\;particles}{weight\;of\;particles}\times 100\%$$

**Figure 4:**
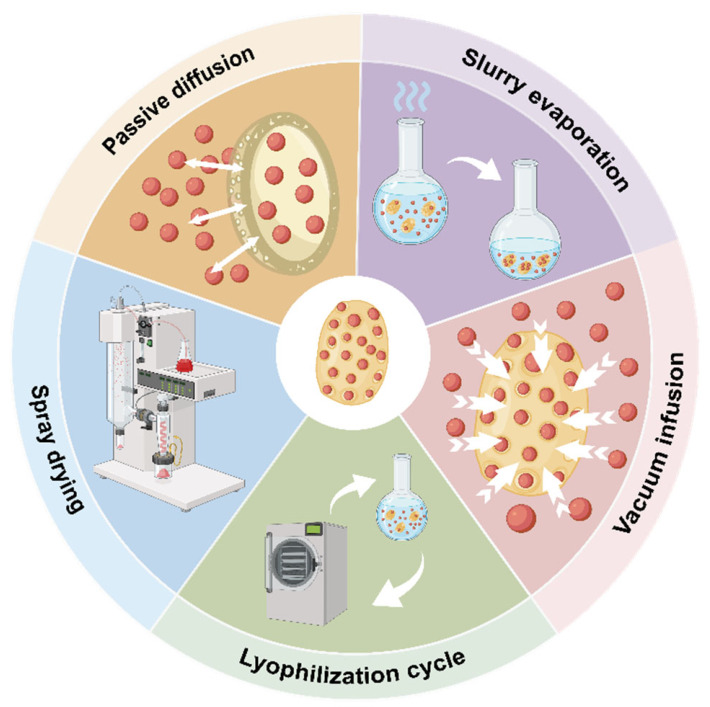
Schematic Diagram of YGPs’ Drug-Loading Method.

Selecting an appropriate loading strategy based on the physicochemical properties of drug molecules (such as solubility, molecular size, and stability) is crucial for enhancing the EE and LC of YGPs. Current mainstream loading methods (Figure 4) primarily include passive diffusion, slurry evaporation/pH-driven method, vacuum infusion, freeze-drying cycles and spray drying.

### 4.1. Passive Diffusion

Passive diffusion remains the most fundamental and widely applied method for preparing drug-loaded YGPs. This technique leverages the principle of concentration gradients by suspending dried or pre-swollen YGPs in a high-concentration drug solution. Driven by the concentration gradient, drug molecules permeate through the porous glucan network on the YGP surface into the internal cavities until equilibrium is reached between the drug concentrations inside and outside the YGPs [51,52,53,54,55]. The advantages of this method include simple operation, mild conditions, and the absence of complex chemical reagents, which significantly preserve drug bioactivity. However, its limitations are also evident: First, EE is relatively low because the drug solution relies solely on concentration gradient-driven free diffusion, resulting in most drugs remaining in the external solution. A study by Šalamúnová et al. demonstrated that hydrophobic and macromolecular drugs achieve an EE of only 20–40% within YGPs via passive diffusion [7]. Second, the absence of a drug “anchoring” mechanism allows the encapsulated drug to reverse-diffuse out during washing or dilution, causing a burst release effect. A study by Dadkhodazade et al. demonstrated that the diffusion behavior of drugs loaded in yeast microcapsules in PBS follows a Fickian mechanism. This further confirms that while the carrier maintains excellent structural integrity under physiological conditions, its drug release relies exclusively on concentration gradient-driven pore diffusion [56]. Consequently, this method is typically only suitable for small-molecule drugs with good water solubility that can form electrostatic adsorption with the glucan matrix.

### 4.2. Slurry Evaporation/pH-Driven Method

To overcome the issues of low drug-loading capacity and reverse permeation in passive diffusion methods, researchers developed an in situ precipitation strategy leveraging differences in drug solubility across various solvents or pH environments. This approach aims to address the challenge of loading hydrophobic drugs. The strategy primarily comprises solvent evaporation and pH-driven precipitation methods. The solvent evaporation method dissolves hydrophobic drugs in organic solvents (e.g., ethanol, acetone). After the drug solution permeates into the YGPs, the solvent is removed via vacuum distillation or natural evaporation. As the solvent diminishes, the drug reaches a supersaturated state within the YGP cavities and precipitates out [57,58], achieving drug loading into the YGPs. The pH-driven precipitation method utilizes drugs with pH-dependent solubility (e.g., amphotericin B, curcumin). Adsorption occurs at a pH where drug solubility is high, followed by rapid adjustment of the pH to the drug’s insoluble range. This induces a phase transition within the microcapsule, causing the drug to deposit within the cavity [8].

This method significantly enhances the drug-loading capacity for hydrophobic drugs, and the resulting solid precipitate enables sustained-release effects by delaying drug release. However, it is important to note that residual organic solvents and drastic pH changes may affect the activity of protein-based drugs.

### 4.3. Vacuum Infusion

Vacuum infusion is a strategy that utilizes physical pressure differentials for drug loading. During the loading process, the drug solution is first blended with YGPs, followed by vacuum treatment. Under pressure, the drug solution is forcibly “pressed” into the hollow internal structure of the YGPs [59,60]. Research by Young et al. demonstrated that compared to passive diffusion, vacuum infusion increased the encapsulation efficiency of curcumin by 3-fold and that of fisetin by 2-fold, while reducing the required time by 288-fold [61], thereby enhancing drug-loading efficiency.

### 4.4. Freeze-Drying Cycles

The freeze-drying method involves mixing dry YGPs with a high-concentration drug solution, allowing the drug and solvent to permeate the β-glucan shell. Upon freeze-drying, the solvent is removed through sublimation, precipitating the non-volatile drug within the YGP cavities. To overcome the low drug loading typical of a single cycle, the cyclic freeze-drying method achieves layered drug accumulation by repeatedly performing the “liquid absorption-freeze-drying” operation until the desired loading capacity is reached. [54,62].

### 4.5. Spray Drying

Spray drying is an efficient method for preparing drug-loaded microcapsules, suitable for large-scale production. This technique atomizes a mixture of drug and encapsulation material into fine droplets, which are then rapidly dried to form microcapsules. Dadkhodazade et al. compared the effects of different drying methods on the EE of cholecalciferol in yeast carriers [56]. Results indicated that the yeast-based system prepared via spray drying achieved a high EE (76.10 ± 6.92%). However, the requirement for specialized equipment limits the widespread application of this method.

## 5. Advantages of YGPs in Oral Drug Delivery Systems

In recent years, although traditional polysaccharide-based carriers—such as chitosan and alginate—have been extensively investigated for oral delivery, their structural instability within the complex GI tract remains a critical bottleneck. These carriers typically rely on physical cross-linking to form hydrogel matrices; however, when exposed to the extreme pH fluctuations of the GI environment, these highly hydrated networks are highly susceptible to over-swelling, which leads to rapid structural disintegration. This swelling-induced barrier failure directly results in severe premature drug leakage, with burst release rates in simulated gastric fluid (SGF) frequently reaching as high as 30–50% [63,64].

In stark contrast, YGPs exhibit superior resistance to degradation and remarkable mechanical stability. Their unique natural shell architecture enables YGPs to maintain an intact hollow morphology even in highly acidic environments. Leveraging this robust structural integrity, YGPs successfully transit the gastrointestinal barrier and are transported via M cells into the GALT. Subsequently, they are specifically recognized and efficiently internalized by intestinal macrophages via Dectin-1 receptors, thereby exerting immunomodulatory effects alongside precise drug delivery.

Consequently, YGPs represent a highly promising and versatile platform for achieving precise and stable oral drug delivery. The specific functional advantages over conventional carriers are systematically elaborated in the following aspects.

### 5.1. Hollow Structure for Loading Drugs

**Figure 5:**
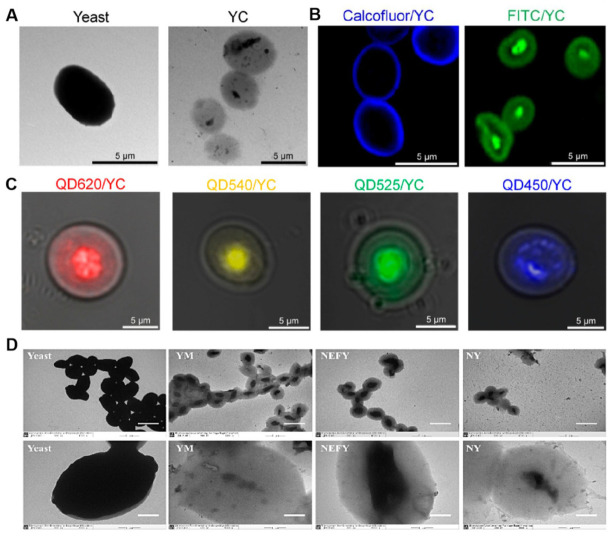
Structural characterization, encapsulation potential, and gastrointestinal stability of YGPs. (**A**) Transmission electron microscopy (TEM) images of intact yeast (left) and YGPs (right) prepared by serial alkaline and solvent extractions. (**B**) fluorescence staining of YGPs with calcofluor white (left, blue) or FITC (right, green) showing the typical capsular structure. (**C**) The hollow cavity allows for the encapsulation of diverse nanoparticles, illustrated here by the loading of various Quantum Dots (QDs) as model drugs [69]. Copyright © 2017 American Chemical Society. (**D**) TEM micrographs of yeast, Yeast Microcapsules, Natural antioxidant nobiletin (NOB)-loaded yeast microcapsule (NEFY), and NY. NY was NOB loaded in YM without metal polyphenol network (MPN) [14]. Copyright © 2024, American Chemical Society.

One of the significant advantages of YGPs is their unique hollow structure (Figure 5A,B). By removing the cytoplasmic components of yeast cells, the retained cell wall skeleton forms micron-sized particles with an internal cavity [39,65,66,67,68]. This natural “container” provides an ideal space for drug encapsulation, enabling efficient loading of various hydrophilic or hydrophobic drugs, including small molecules (Figure 5C), proteins, peptides, nucleic acids, and even nanoparticles [69] (Figure 5D).

YGPs also offer physical protection to their encapsulated drugs. Oral medications face harsh conditions during gastrointestinal transit, including strong gastric acidity (pH 1–3), pepsin degradation, and attacks by digestive enzymes like pancreatic enzymes and bile salts in the small intestine [1]. The primary component of YGPs, β-glucan, remains undigested in the gastrointestinal tract [70]. Its robust structure acts like “armor,” effectively shielding encapsulated drugs from direct contact with these degradative factors, thereby significantly improving drug stability and bioavailability in the gastrointestinal tract. For instance, Yang et al. demonstrated that YGPs effectively shielded berberine from gastrointestinal degradation [39]. Chitosan-coated YGPs developed by Mohammed et al. exhibited excellent gastrointestinal stability, releasing only approximately 60% of cefuroxime within 24 h under simulated intestinal pH conditions [35]. Studies by Li et al. [15] and Han et al. [16] also indicate that YGPs can effectively protect encapsulated nanoparticles and drugs, enhancing their stability in the gastrointestinal tract.

Compared to traditional hollow carriers like liposomes or polymer microspheres, YGPs possess unique advantages in delivering poorly soluble and cytotoxic drugs. For instance, Šalamúnová et al. discovered that YGPs could load curcumin in an amorphous form, increasing its dissolution rate by fourfold [7]. Research by He et al. demonstrated that YGPs effectively overcome the application limitations of avenanthramide-c (AVN-C) due to its poor solubility, highlighting their potential for enhancing the bioavailability of poorly soluble drug [57]. Concurrently, He et al.’s work demonstrated that delivering the antitumor drug doxorubicin (DOX) using YGPs with a hollow porous vesicle structure effectively reduced the drug’s inherent cytotoxicity [71], offering a novel approach for delivering cytotoxic drugs.

### 5.2. Targeted Delivery

Another advantage of YGPs in oral drug delivery systems is their inherent cellular targeting capability. This is primarily due to the β-glucan component of YGPs, which can be specifically recognized and phagocytosed by immune cells in the gut. β-glucan is aptly described as a “compass” for drugs, guiding them to precisely reach target sites [18].

**Figure 6:**
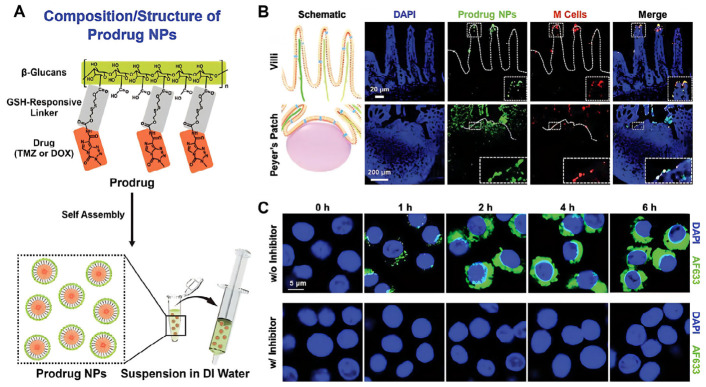
Yeast β-glucan enables specific receptor targeting and M-cell transport. (**A**) Schematic illustration of the prodrug construction. (**B**,**C**) prodrug NPs had been pre-labeled with Alexa Fluor 633 (f-prodrug NPs). (**B**) In vivo targeting to M cells in Peyer’s patches. (**C**) Competitive inhibition assay. CLSM images showing the uptake of f-prodrug NPs by RAW264.7 macrophages. Pre-treatment with laminarin, a Dectin-1 inhibitor, effectively blocked the entry of nanoparticles, confirming that uptake is dependent on Dectin-1 recognition [74]. Copyright © 2021 Wiley-VCH GmbH.

This targeting is achieved through two main mechanisms: (1) M cell-mediated transport: Peyer’s patches (PPs) constitute a vital component of GALT, whose surfaces are covered by M cells. M cells possess highly efficient antigen uptake and transport capabilities, enabling them to shuttle particulate matter from the intestinal lumen into the underlying lymphoid tissue to initiate immune responses [20,72]. Studies indicate that β-1,3-glucan can be recognized and internalized by M cells (Figure 6B), facilitating the transport of YGPs and their drug payloads into GALT [18,20,26]. This process is crucial for oral vaccine delivery and intestinal immune modulation. (2) Dectin-1 receptor-mediated immune cell targeting: Dectin-1 belongs to the C-type lectin family and is a classic type II transmembrane protein. It is primarily expressed on immune cells such as macrophages, neutrophils, and dendritic cells. β-glucan can specifically bind to Dectin-1 and Toll-like receptors highly expressed on the surface of macrophages and other phagocytes, thereby achieving targeted interaction with immune cells [73] (Figure 6C).

By leveraging this specific recognition, YGPs are first transferred to PPs via M cells, then specifically taken up by immune cells aggregated within PPs, and subsequently transported by immune cells to distant pathological sites [74].

### 5.3. Immune Modulation

β-glucan is a potent immunostimulant recognized as a pathogen-associated molecular pattern (PAMP) by host PRRs, particularly Dectin-1 and complement receptor 3 (CR3) [75]. Binding triggers pathways including PI3K/Akt/mTOR and MAPK/NF-κB [76,77], inducing the secretion of pro-inflammatory cytokines such as TNF-α, IL-6, and IL-12 [78,79].

**Figure 7:**
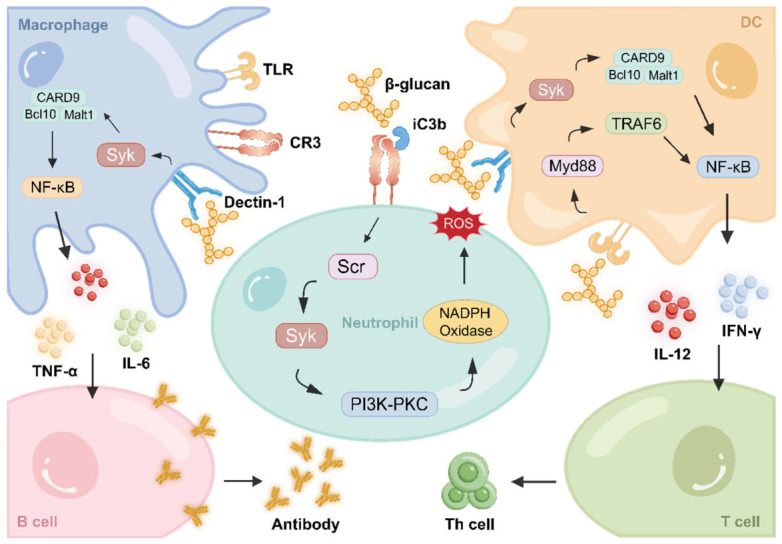
Schematic Diagram of β-Glucan’s Immunomodulatory Effects.

Macrophages are the primary target cells. Orally ingested particulate β-glucan is transcytosed by M cells to Peyer’s patches, captured by macrophages via Dectin-1, and transported to peripheral immune organs to prime systemic responses [80,81,82]. Notably, β-glucan also induces epigenetic reprogramming in monocytes/macrophages, enabling a heightened response to secondary infections—a phenomenon termed “trained immunity” [83]. Beyond macrophages, β-glucan modulates other immune cells to bridge innate and adaptive immunity [84,85] (Figure 7). In dendritic cells (DCs), binding to Dectin-1 activates Syk-dependent signaling and the Dectin-1-CARD9 axis, which upregulates MHC class II and co-stimulatory molecules to enhance antigen presentation. Concurrently, crosstalk between Dectin-1 and TLR2/4 signaling promotes the secretion of Th1-polarizing cytokines (IL-12, IFN-γ) [86,87]. For neutrophils, which primarily rely on the CR3 receptor rather than Dectin-1 [88,89], β-glucan binds to the CR3 lectin site. This binding, often in synergy with the complement fragment iC3b, triggers an activated state that primes rapid reactive oxygen species (ROS) release, degranulation, and CR3-dependent cellular cytotoxicity against tumors [90]. Furthermore, β-glucan indirectly promotes T cell proliferation [91], Th1 differentiation [92,93,94], and B cell antibody production (e.g., sIgA) [95,96,97,98,99], making it a highly effective mucosal vaccine adjuvant.

### 5.4. Safety

The application of YGPs as oral drug delivery carriers hinges on their excellent biosafety, a pivotal prerequisite for clinical translation. Saccharomyces cerevisiae, a microorganism extensively used in food fermentation and biopharmaceuticals, is designated as GRAS by the U.S. FDA [24]. This designation implies that yeast and its microcapsule derivatives exhibit negligible toxicity and minimal risk of adverse events under standard usage conditions.

The biosafety of YGPs is primarily manifested in the following aspects:

#### 5.4.1. Biocompatibility

The yeast cell wall primarily consists of natural polysaccharides such as β-glucan and mannan, which demonstrate excellent biocompatibility in the human body and are unlikely to elicit severe immune rejection or allergic reactions [24]. Multiple studies have confirmed the excellent biocompatibility of YGPs and their composites in both in vivo and in vitro experiments [33].

#### 5.4.2. Biodegradability

The natural polysaccharide components of YGPs can be degraded in vivo by enzymes such as β-glucanase. The resulting SCFAs produced during colonic fermentation also function as prebiotics, promoting the growth and proliferation of beneficial bacteria while precluding the risk of bioaccumulation [17]. This degradability is crucial for drug delivery systems, ensuring carriers are efficiently cleared after completing their delivery function.

#### 5.4.3. Inherent Advantages of Natural Origin

Compared to synthetic polymers or inorganic nanomaterials, YGPs—as a natural biomaterial—typically involve more eco-friendly production processes and reduce the risk of introducing harmful impurities during synthesis. This natural attribute also enhances public acceptance, facilitating future clinical translation.

Despite the favorable biosafety profile of YGPs, immune responses potentially elicited by the capsules themselves warrant attention. Furthermore, when developing composite YGP systems, rigorous safety assessments of introduced composite materials remain essential to ensure the biocompatibility and low toxicity of the entire delivery system. For instance, when YGPs are engineered with materials such as chitosan, alginate, metal–phenolic networks (MPNs), or inorganic nanoparticles, comprehensive assessments of the composite material’s biosafety are required [100,101]. The inherent safety of YGPs as natural carriers provides a solid foundation for their extensive application in oral drug delivery.

In summary, YGPs have been extensively utilized in oral drug delivery systems due to their natural hollow structure, which provides an ideal encapsulation compartment for drug loading. This structure shields therapeutics from the harsh gastric environment characterized by low pH and pepsin-mediated degradation, while concurrently exhibiting excellent targeting capabilities, immunomodulatory activity, and biosafety.

## 6. Limitations of Yeast Microcapsules and Improvement Strategies

Despite the numerous advantages demonstrated by yeast microcapsules in oral drug delivery systems, YGPs also exhibit certain limitations, making it challenging to fully meet intricate clinical demands of drug delivery. Consequently, researchers are actively exploring the introduction of composite materials to overcome these issues, thereby enhancing the stability and functionality of YGPs to achieve superior drug delivery and therapeutic outcomes.

### 6.1. Limitations of Yeast Microcapsules

YGPs, consisting solely of the yeast cell wall β-glucan as a carrier, possess inherent protective and targeting capabilities. Nevertheless, they exhibit limitations in the following aspects: (1) Drug leakage due to porous structure: After removing intracellular components, the YGPs a porous structure. While this facilitates drug encapsulation, it may also lead to premature leakage of encapsulated drugs, particularly in complex environments like the gastrointestinal tract [18]. For drugs requiring prolonged sustained release or precise release at specific sites, the release control capability of YGPs may be insufficient. For this reason, YGPs are often combined with other systems when used as carriers for orally targeted formulations [102]. (2) Limited Functional Versatility: YGPs rely primarily on the intrinsic properties of β-glucan, resulting in a lack of functional diversity. They often lack the requisite versatility to meet complex therapeutic demands, such as multi-stimuli responsiveness (e.g., to pH, temperature, enzymes, or redox potential), For complex therapeutic demands requiring multi-stimulus responsiveness (e.g., pH, temperature, enzymes, redox potential), multi-drug synergistic delivery, or more precise targeting, YGPs alone often fall short. For instance, they may fail to achieve precise responses to varying pH levels within the gastrointestinal tract or simultaneously deliver two drugs with different physicochemical properties.

### 6.2. Improvement Strategies

To overcome the limitations of YGPs, researchers commonly employ a strategy of combining them with other functional materials to construct composite YGP systems. This composite approach aims to enhance the protective capacity, precise release control, targeting efficiency, and multi-stimulus responsiveness of YGPs through synergistic interactions between YGPs and the incorporated materials.

Currently, strategies for functionalizing and optimizing YGPs can be broadly categorized into the following four approaches (Table 1).

#### 6.2.1. Nanoparticle Encapsulation Strategy

To mitigate the challenge of premature drug release caused by the rapid diffusion of small molecules through the YGPs, researchers have proposed a strategy of integrating YGPs with nanoparticles. This involves the pre-encapsulation of therapeutics into nanoparticles and then loading them into the interior of YGPs [103,104,105,112]. This approach effectively prevents premature leakage by leveraging the steric hindrance and electrostatic interactions of the nanoparticles.

In a representative study, Zhang et al. engineered a composite system using mesoporous polydopamine (MPDA) nanoparticles as the core carrier. These nanoparticles were surface-functionalized with manganese dioxide (MnO_2_) nanozymes and loaded with diallyl trisulfide (DATS), a precursor for the gasotransmitter hydrogen sulfide (H_2_S). Subsequently, YGPs were utilized to biomimetically camouflage this composite nanocore. The MPDA core utilizes its high adsorption capacity to retain small-molecule drugs, effectively precluding their leakage through the YGP pores [103]. Furthermore, the incorporation of nanostructures not only mitigates premature drug leakage but also endows the delivery platform with stimuli-responsive release profiles. For instance, Han et al. developed a hierarchical composite delivery system termed “yeast-shell-encapsulated supramolecular nanoparticles” (Man-CUR NYPs) [16]. This system leverages the host-guest interaction between β-cyclodextrin and adamantane to construct functionalized curcumin nanoparticles, which are subsequently sequestered within YGPs via electrostatic adsorption and vacuum extrusion techniques. The resulting platform exhibits dual sensitivity to ROS and pH. Wang et al. similarly utilized YGPs as a protective outer shell to design a delivery system with microenvironment-responsive release [104]. The inner core of this system consisted of EMO-loaded nanoparticles, which were fabricated through the disulfide-crosslinking of hyaluronic acid-rhein conjugates. By encapsulating these redox-responsive polymeric nanoparticles within the YGPs, the researchers achieved a robust composite structure that resisted gastrointestinal degradation while enabling microenvironment-triggered payload release in the inflamed colon. Focusing on the delivery of the same small-molecule drug, EMO, Pu et al. engineered a yeast-based delivery system featuring dual-targeting layers [112]. By utilizing lactoferrin (Lf) as a carrier to encapsulate the hydrophobic drug EMO into nanoparticles (EMONPs) and subsequently sequestering them within YGPs, this system achieved precise accumulation and multi-stage delivery of the drug within inflamed colonic tissues, thereby significantly augmenting the anti-inflammatory therapeutic efficacy.

**Figure 8:**
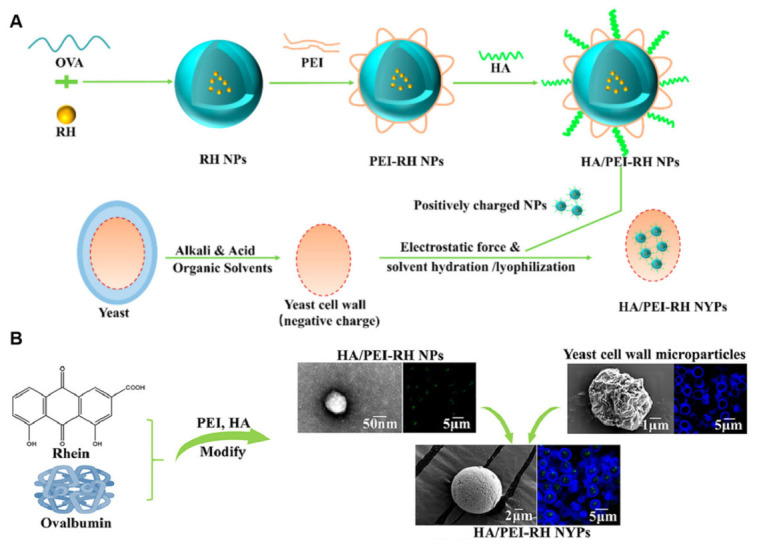
Design and Characterization of HA/PEI-RH NYPs. (**A**) Schematic illustration of the step-by-step fabrication process of HA/PEI-RH NYPs. (**B**) SEM of HA/PEI-RH NPs and SEM of HA/PEI-RH NYPs [17]. Copyright © 2021 American Chemical Society.

To address the poor water solubility and premature leakage associated with rhein, Chen et al. developed a dual-targeting oral delivery system featuring a ‘core-shell’ architecture [17]. In this design, rhein was encapsulated within ovalbumin (OVA) nanoparticles, which were surface-functionalized with polyethyleneimine (PEI) and hyaluronic acid (HA) (Figure 8). Subsequently, these functionalized nanoparticles were sequestered within the hollow cavity of YGPs via electrostatic deposition. This hierarchical structure not only effectively precluded drug leakage during gastrointestinal transit but also integrated the M-cell targeting ability of YGPs with the CD44 receptor-specific recognition of HA.

Remarkably, the application of such hierarchical architectures is not limited to therapeutic delivery, but also extends to in vivo detoxification. For instance, Hamza et al. developed a yeast cell wall-based micro-nano composite adsorbent to overcome the difficulty of removing aflatoxin B_1_ (AFB_1_) in vivo [105]. By encapsulating humic acid nanoparticles—known for their strong toxin adsorption capacity—within YGPs, the researchers leveraged the biocompatibility of the YGPs while significantly enhancing the specific surface area and adsorption efficiency of humic acid through nanoscale encapsulation.

In summary, the strategy of pre-encapsulating small-molecule therapeutics into nanoparticles prior to their loading within YGPs effectively circumvents the premature leakage caused by the intrinsic porous architecture of YGPs, thereby enabling precise drug delivery to the pathological site and achieving superior therapeutic outcomes.

#### 6.2.2. Metal–Phenolic Network Assembly Strategy

MPNs are constructed by leveraging the rapid supramolecular coordination between natural polyphenols (e.g., tannic acid [TA], epigallocatechin gallate [EGCG]) and metal ions (e.g., Fe^3+^, Zn^2+^, Al^3+^). This process generates functionalized thin films within the interior of YGPs, which not only serve to stably encapsulate the drug payload within the hollow cavity but also endow the delivery system with potent antioxidant properties. The intrinsic antioxidant activity of these networks originates from the abundant phenolic hydroxyl groups present in the polyphenol precursors. These groups function as highly efficient electron or hydrogen atom donors to rapidly scavenge deleterious ROS. Furthermore, the incorporation of transition metal ions is pivotal; they act not only as supramolecular cross-linkers that rapidly assemble soluble polyphenols into a robust physical barrier to prevent drug leakage, but also as stimuli-responsive nodes that impart pH-triggered release capabilities and intrinsic nanozyme-like catalytic activities to the delivery system [113].

Representing the paradigm of iron-based coordination, Li et al. engineered a novel drug delivery system utilizing a MPN formed via the coordination of EGCG and Fe^3+^ [15]. The anti-inflammatory polyphenol curcumin (Cur) was encapsulated within this MPN to generate Cur-MPN, which was subsequently sequestered within YGPs. This MPN architecture demonstrated potent antioxidant activity while effectively preventing the premature leakage of the small-molecule payload. Validating the broad applicability of this EGCG-based strategy, Feng et al. [52] and Yang et al. [14] similarly employed EGCG-based MPNs to efficiently encapsulate small-molecule anti-inflammatory and antioxidant agents within the hollow cavity of YGPs. This strategy enabled precise targeting of macrophages at inflammatory loci and the regulation of macrophage polarization, thereby effectively treating ulcerative colitis.

**Figure 9:**
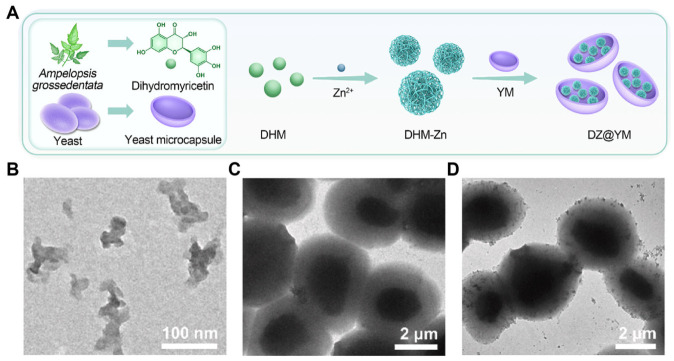
Design and Characterization of DZ@YM. (**A**) Schematic illustration of the step-by-step fabrication process of DZ@YM. (**B**,**C**) TEM images of DHM-Zn (**B**), YM (**C**), and DZ@YM (**D**) [25]. Copyright © 2025 The Authors. Published by Elsevier Ltd.

Expanding beyond iron-centric networks to explore broader coordination chemistry, Chai et al. encapsulated a “metal-phenolic nanozyme”—constructed by coordinating dihydromyricetin (DHM) with zinc ions (Zn^2+^)—within YGPs, successfully obtaining the DZ@YM composite drug delivery system (Figure 9A). Transmission electron microscopy characterization of the three types of particles (DHM-Zn, YM, and DZ@YM) confirmed that the nanozyme was successfully loaded into the YGP cavities (Figure 9B–D). By harnessing the intrinsic anti-inflammatory and antioxidant properties of the nanozyme, this oral delivery system integrated antioxidant defense, inflammation suppression, and gut microbiota restoration for the synergistic treatment of ulcerative colitis [25].

In summary, the integration of metal–phenolic networks not only effectively circumvents the leakage of small-molecule therapeutics but also functionalizes the delivery system with intrinsic antioxidant capabilities.

#### 6.2.3. In Situ Biomineralization Strategy

To augment drug-loading capacity and preserve the biomacromolecules (e.g., proteins and vaccines), researchers have exploited the internal hollow cavities of YGPs as “microreactors” to facilitate the in situ formation of inorganic mineral scaffolds or polymeric networks.

Exemplifying the in situ mineralization strategy, Zhang et al. ingeniously utilized metabolic CO_2_ generated during yeast respiration to drive the in situ mineralization of calcium carbonate (CaCO_3_) within the cellular cavity [33]. The resulting mineralized scaffold not only efficiently sequestered the small-molecule drug curcumin but also exhibited superior acid-neutralizing capacity. Similarly leveraging inorganic scaffolds to protect delicate biologics, Soto et al. proposed an “in situ silicification” strategy by employing tetraethyl orthosilicate (TEOS) to grow robust silica nanocages within YGPs [65]. By allowing the infiltration of a soluble TEOS-protein precursor prior to polymerization, this method efficiently immobilizes proteins within the YGP cavity. This confinement significantly enhances the proteins’ thermal stability and resistance to denaturation under extreme conditions.

**Figure 10:**
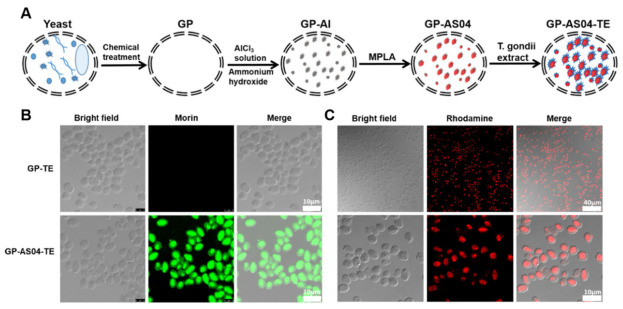
Design and Characterization of GP-AS04-TE particles. (**A**) Schematic illustration of the step-by-step fabrication process of GP-AS04-TE particles. (**B**) GP-AS04-TE and GP-TE particles were stained with morin and observed under a confocal microscope for aluminum detection. (**C**) Rhodamine-labeled TE was absorbed into GP–AS04 particles and observed under a confocal microscopy [106]. Copyright © 2021 American Chemical Society.

Expanding the utility of this in situ mineralization paradigm to immunotherapy, researchers have developed an “in situ alum mineralization” antigen delivery system. By inducing the in situ precipitation of aluminum salts (alum) within YGPs, antigens—such as HBsAg or Toxoplasma antigens—are efficiently adsorbed and physically immobilized via the internal mineral framework [106,107] (Figure 10A). Furthermore, the successful loading of the payload was verified through confocal microscopy; the mineralized aluminum was detected via green fluorescent morin staining, while the loaded *Toxoplasma* extract (TE) was tracked using red fluorescent rhodamine labeling (Figure 10B,C). This hybrid system synergizes the adjuvant effect of alum with the intrinsic targeting capability of YGPs toward antigen-presenting cells (APCs). Consequently, this strategy elicits significantly more potent humoral and cellular immune responses compared to traditional alum adjuvants, underscoring the broad applicability of this strategy in enhancing vaccine immunogenicity.

Beyond the construction of rigid mineral frameworks, the in situ modulation of the internal microenvironment—specifically through hydrophobicity regulation—provides an alternative mechanism for effective payload encapsulation. He et al. employed YGPs as carriers for the delivery of the oat biomarker AVN-C [57]. By leveraging strong hydrophobic interactions and hydrogen bonding between the YGPs and the cargo, they induced a highly hydrophobic state within the capsule interior, thereby achieving efficient physical entrapment. The novelty of this strategy lies in the induction of a critical “high-hydrophobicity transition” within the YGPs during the loading process. This physicochemical alteration effectively shielded the bioactive payload from degradation and enabled prolonged sustained release under simulated gastrointestinal conditions.

#### 6.2.4. Surface Chemical Modification and External Gel Encapsulation Strategy

Functionalization of yeast glucan particles through surface engineering strategies—including chemical grafting, physical coating, and external gel encapsulation—can effectively enhance their drug-carrying capacity and confer specific targeting capabilities.

The covalent conjugation of functional moieties onto the polysaccharide hydroxyl or amino groups of the YGP surface represents a potent strategy to modulate the in vivo pharmacokinetics of YGPs. To surmount the limitations of passive macrophage targeting, He et al. utilized the Schiff base reaction to graft HA onto the YGP surface [71]. The specific recognition of HA for CD44 receptors on tumor cells endowed the carrier with active targeting capabilities against malignancies such as breast cancer. Addressing the challenge that the intrinsic negative surface charge of YGPs hinders the efficient encapsulation of similarly anionic antigens, Yang et al. achieved surface charge reversal via polyethyleneimine (PEI) modification [108]. The resulting cationic PEI-YGPs demonstrated superior adsorption of negatively charged antigens, thereby significantly amplifying the immune response of the oral vaccine. Furthermore, to enhance the water solubility of extracted β-(1,3)-glucans (BYGs) for intravenous administration, Chen et al. synthesized amphiphilic BYG conjugates by grafting hydrophilic polyethylene glycol (PEG). Hydrophobic methotrexate (MTX) was efficiently loaded into the self-assembled nanoparticles formed by these conjugates; upon tail vein injection into rheumatoid arthritis mice, this system effectively improved MTX bioavailability while mitigating systemic side effects [114].

Transitioning from covalent modifications to non-covalent strategies, the deposition of polymer coatings on YGP surfaces via electrostatic interactions or hydrogen bonding serves as a facile and versatile strategy for sealing and protection. Layer-by-Layer (LbL) assembly, a classic “sandwich” encapsulation strategy, involves the alternating deposition of oppositely charged polyelectrolytes or functional materials to construct multilayered films on the YGP surface [109,115,116,117]. This technique allows for precise modulation of film parameters—including layer number, thickness, composition, and functionality—thereby fine-tuning drug release kinetics and targeting profiles. Crucially, the integration of smart polymer coatings into these LbL assemblies endows the system with pH-responsive release capabilities. By maintaining a compact, impermeable structure in the acidic gastric environment, these coatings shield the payload from degradation. Upon reaching the neutral pH of the intestine, the multilayered film undergoes controlled swelling and structural relaxation, triggering the sustained release of the drug [118]. For instance, Tan et al. utilized cationic chitosan (CS) and anionic chondroitin sulfate to construct alternating layers on anthocyanin-loaded YGPs, significantly enhancing encapsulation efficiency and retention rates [109]. This multilayered architecture not only effectively seals surface pores but also regulates intestinal release rates via layer number adjustment. Beyond complex LbL architectures, simpler polymer coatings also offer substantial protective efficacy. Coating YGPs with chitosan or oleic acid-modified chitosan (CSO) leverages the pH-sensitivity of chitosan (insoluble in acidic gastric fluid, soluble in neutral pH) to shield internal payloads from gastric degradation while enhancing carrier hydrophobicity and stability [34,35]. Additionally, exploiting the coacervation between xanthan gum and yeast cells can form a dense protective membrane suitable for encapsulating water-soluble bioactives [119].

Finally, to provide robust protection for sensitive biologics (e.g., insulin) or probiotics, researchers have adopted a “Matryoshka-like” hierarchical macro-encapsulation strategy, wherein drug-loaded YGPs are further entrapped within larger hydrogel microbeads. Examples include incorporating YGPs into sodium alginate/whey protein beads [110] or chitosan/sodium alginate microgels [111]. In such hierarchical systems, the external hydrogel matrix serves as the primary defense barrier against gastric acid erosion and controls overall swelling, while the internal YGPs function as the secondary defense responsible for targeted transport. These hydrogel-based encapsulation platforms also exhibit unique pH-responsive release kinetics. For instance, Hou et al. demonstrated that in simulated gastric fluid, the microgels swell to only 200% of their original volume, maintaining a compact structure that protects the encapsulated payload from gastric degradation. Conversely, in the intestinal environment, the swelling ratio reaches 3000%, and the weakening of electrostatic interactions and the disintegration of the gel network enable the sustained release of the drug [111]. This strategy is particularly advantageous for complex oral delivery systems designed for insulin or probiotic therapy, drastically preserving the bioactivity of macromolecules and enhancing their oral bioavailability.

## 7. Applications of YGPs in Treating Various Diseases

YGPs exhibit substantial potential as oral drug delivery platforms, owing to their distinct biological and structural attributes. With inherent targeting properties, immunomodulatory activity, and superior drug shielding efficacy, they are attractive vehicles for the management of inflammatory diseases and malignancies, and serve as effective vaccine carriers.

### 7.1. Inflammatory Diseases Treatment

#### 7.1.1. Inflammatory Bowel Disease (IBD)

IBD, encompassing Crohn’s disease and ulcerative colitis (UC), is a chronic, recurrent inflammatory condition of the intestines. Current treatments face challenges such as significant systemic side effects and poor targeting efficacy. YGPs have emerged as promising therapeutic carriers for IBD due to their natural targeting affinity for Peyer’s patches and the resident immune cells. β-glucans are recognized and internalized by M cells in Peyer’s patches and macrophages enriched at inflammatory sites, enabling precise drug delivery to inflamed tissues.

**Figure 11:**
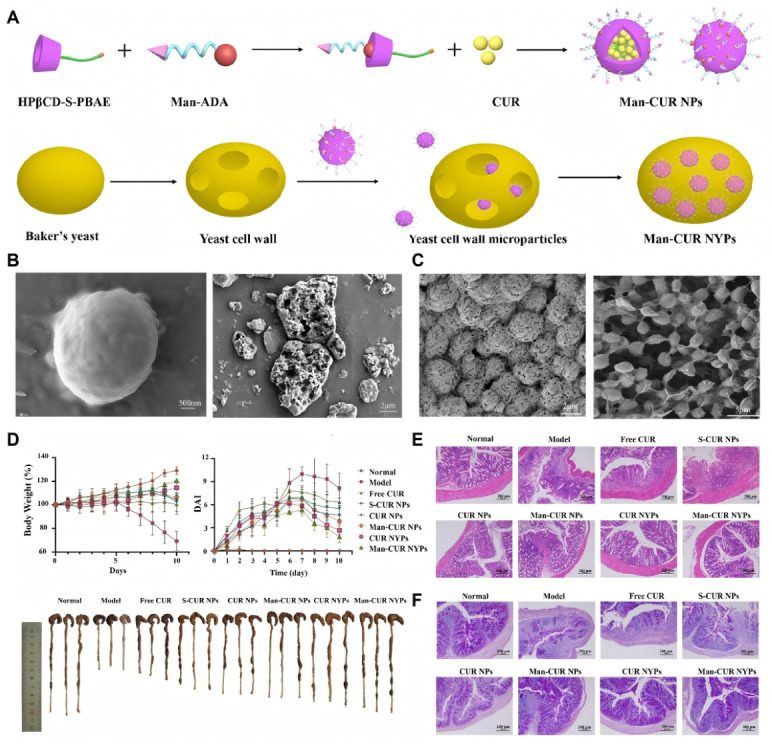
Fabrication and therapeutic evaluation of targeted yeast microcapsules (Man-CUR NYPs) for the treatment of ulcerative colitis. (**A**) Schematic illustration of the step-by-step preparation process of Man-CUR NYPs (**B**) Stability evaluation of NYPs. (**C**) SEM image of YPs and Man-CUR NYPs. (**D**) In vivo therapeutic efficacy in a colitis mouse model, indicated by body weight changes, Disease Activity Index (DAI) scores, and representative macroscopic photographs of harvested colons across different treatment groups. (**E**) H&E-stained and (**F**) PAS-stained histopathological sections of the colonic tissues [16]. under the Creative Commons CCBY 4.0 license.

Multiple studies have demonstrated the therapeutic efficacy of YGPs in IBD. Utilizing the CM@YM delivery platform, Li et al. achieved targeted accumulation in inflamed colonic tissues. Upon oral administration in UC models, this system successfully scavenged localized ROS, regulated macrophage polarization toward the anti-inflammatory phenotype, and effectively restored gut microbiota diversity [15]. Similarly, Han et al. developed “dual-sensitive supramolecular curcumin nanoparticles” encapsulated within “advanced yeast particles” (Man-CUR NYPs) to synergistically treat UC (Figure 11A). Morphological characterization via scanning electron microscopy (SEM) confirmed the successful preparation of the composite drug delivery system (Figure 11B,C). Through comprehensive in vivo evaluations in a colitis mouse model, the authors demonstrated the remarkable therapeutic efficacy of this system against inflammatory bowel disease. Specifically, oral administration of Man-CUR NYPs effectively prevented body weight loss, reduced the Disease Activity Index (DAI), and significantly alleviated colon shortening (Figure 11D). Furthermore, histological analyses utilizing H&E and PAS staining verified that this targeted delivery system successfully ameliorated colonic inflammation, mitigated tissue damage, and promoted mucosal barrier restoration by modulating macrophage reprogramming and scavenging ROS (Figure 11E,F) [16]. Furthermore, Chen et al. encapsulated macrophage-targeted nanoparticles within yeast particles (YPs) [17]. Drug release was triggered by β-glucanase-mediated degradation in the colon, with HA ligands enhancing macrophage targeting. This approach effectively inhibited the TLR4/MyD88/NF-κB pathway and alleviated inflammation. Yang et al.’s NEFY system significantly accumulated in colonic macrophages, attenuating oxidative stress, inhibiting the NLRP3 inflammasome, and promoting mucosal repair [14].

Collectively, these studies indicate that YGPs and their composite systems hold substantial potential for IBD treatment. Through multiple mechanisms—including targeted delivery, immunomodulation, antioxidant effects, and gut microbiota regulation—they effectively alleviate inflammation and facilitate intestinal repair.

#### 7.1.2. Arthritis

Arthritis is a chronic inflammatory disease characterized primarily by synovial inflammation and cartilage degeneration. Driven by multiple factors, including immune imbalance and metabolic disorders, it ultimately leads to joint structural destruction and loss of function. Clinically, arthritis is classified into subtypes such as osteoarthritis (OA), rheumatoid arthritis (RA), and septic arthritis based on etiology. Among these, RA represents a highly disabling autoimmune disease that severely compromises patient quality of life, posing a significant challenge in current clinical management. Within the RA microenvironment, macrophages are widely recognized as pivotal effector cells driving the inflammatory cascade. The chemokines and pro-inflammatory cytokines they secrete accelerate disease progression, implying that modulating macrophage phenotypes is a key therapeutic strategy [120].

To address this, yeast β-glucan-based biomimetic delivery systems have garnered significant attention due to their unique immunotargeting properties. Chen et al. engineered a yeast β-glucan nanocarrier loaded with methotrexate (MTX) that facilitates efficient macrophage uptake and induces polarization from pro-inflammatory M1 to anti-inflammatory M2 phenotypes, thereby effectively suppressing RA inflammation [114]. Moreover, post-traumatic osteoarthritis (PTOA), a classic degenerative cartilage disease, is similarly driven by macrophage-mediated inflammatory responses [121,122]. The utilization of yeast microcapsules (YMPs) as vehicles for gene therapeutics enables precise modulation of the joint immune microenvironment. Zhang et al. demonstrated that YMPs effectively protect the loaded miR365 antagonist during gastrointestinal transit [123]. Following oral administration, the microcapsules are transported via macrophages to the joint site, where the miR365 antagonist is released to downregulate inflammatory and chondrodegenerative signaling pathways, demonstrating the feasibility of treating PTOA via an oral route.

#### 7.1.3. Atherosclerosis

Atherosclerosis (AS) is a pathological process initiated by dysregulated lipid metabolism and driven by vascular endothelial injury and chronic inflammatory responses. Its hallmarks include subintimal lipid deposition, macrophage foam cell formation, and fibrous plaque development, ultimately leading to arterial wall stiffening, lumen narrowing, or rupture [124,125]. Macrophages, which engulf excess cholesterol to form foam cells, are pivotal in promoting plaque formation. They also secrete and respond to various cytokines and chemokines, perpetuating chronic inflammation and tissue damage within the arterial wall. Consequently, targeting macrophages has emerged as a promising nanotherapeutic strategy [126,127,128,129]. Capitalizing on the inherent macrophage-targeting capability of yeast microcapsules, Zhang et al. developed a yeast-derived microcapsule-mediated biomimetic therapy for cardiovascular diseases [130]. This system delivers nanodrugs to Peyer’s patches in the intestine, where they are internalized by resident macrophages and subsequently transported to lesion sites via the lymphatic system. The study demonstrated favorable therapeutic outcomes, highlighting the potential of this approach for treating atherosclerosis and other vascular diseases. Similarly, Yin et al. developed bindarit (a specific synthetic inhibitor of monocyte chemoattractant protein-1)-loaded yeast microcapsules (BIN/YCs) for the immunotherapy of AS [131]. Their results indicated that oral administration of BIN/YCs effectively suppressed atherosclerotic plaque formation by reducing monocyte recruitment to the plaques.

In summary, YGPs-based oral delivery systems offer a promising platform for the management of atherosclerosis.

### 7.2. Cancer Treatment

In cancer therapy, YGPs primarily function as vaccine adjuvants, harnessing their immunomodulatory activity, and as targeted drug carriers, specifically aimed at tumor-associated macrophages (TAMs).

β-glucans can activate the innate immune system, thereby enhancing the host immune response to tumor antigens. Hou et al. incorporated mouse colorectal cancer lysate and the TLR9 agonist CpG into YGPs [28]. They demonstrated that this vaccine elicited robust antibody responses, Th1- and Th17-biased cellular immunity, and immune memory, resulted in significant inhibition of tumor growth. Kiran et al. further highlighted the potential of β-glucan as a cancer biotherapeutic within immunomodulatory and nanotherapeutic strategies [31].

In the context of chemotherapy, YGPs can encapsulate chemotherapeutic agents, utilizing their inherent targeting properties to deliver payloads specifically to tumor sites or tumor-associated immune cells. This approach substantially mitigates off-target toxicity and minimizes systemic adverse reactions. Rajabi et al. synthesized DOX-loaded yeast glucan nanoparticles, demonstrating their ability to inhibit C26 colorectal cancer cell proliferation and promote apoptosis via modulation of the Wnt/β-catenin signaling pathway [51].

**Figure 12:**
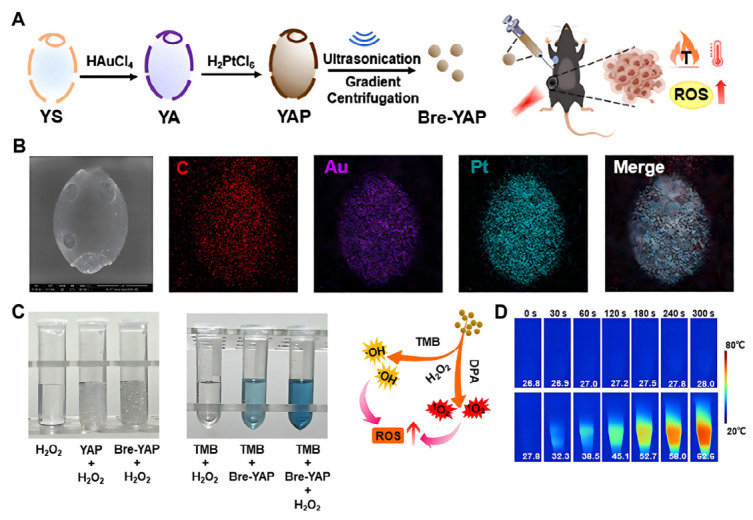
Yeast cell wall fragments functionalized with Au/Pt nanozymes for combined photothermal and chemodynamic cancer therapy. (**A**) Engineering of the Bre-YAP system. Yeast shells serve as a biotemplate for the in situ growth of Au and Pt nanoparticles, followed by fragmentation to enhance tumor penetration. (**B**) Elemental analysis verifies the successful loading of dual-metal nanozymes. (**C**,**D**) The system demonstrates robust ROS generation (**C**) and photothermal conversion capabilities (**D**) [30]. Copyright © 2023 American Chemical Society.

Furthermore, YGPs can be integrated with other therapeutic modalities to achieve synergistic anticancer effects. Zhang et al. developed gold/platinum nanoparticles derived from YGPs for synergistic chemo-photothermal cancer immunotherapy (Figure 12A,B). These nanoparticles preserved the adjuvant activity of yeast glucan while exhibiting superior chemotherapeutic and photothermal efficacy [30] (Figure 12C,D).

### 7.3. IgA Nephropathy Treatment

IgA Nephropathy (IgAN) is a prevalent autoimmune renal disorder whose pathogenesis is intrinsically linked to gut microbiota dysbiosis and mucosal immune abnormalities. Recent research has underscored the critical role of the gut-kidney axis in IgAN, identifying ileal Peyer’s patches as a key inductive site for pathogenic IgA production. In this context, YGPs offer a novel therapeutic strategy by modulating the gut-kidney axis via targeted delivery to M cells in Peyer’s patches.

**Figure 13:**
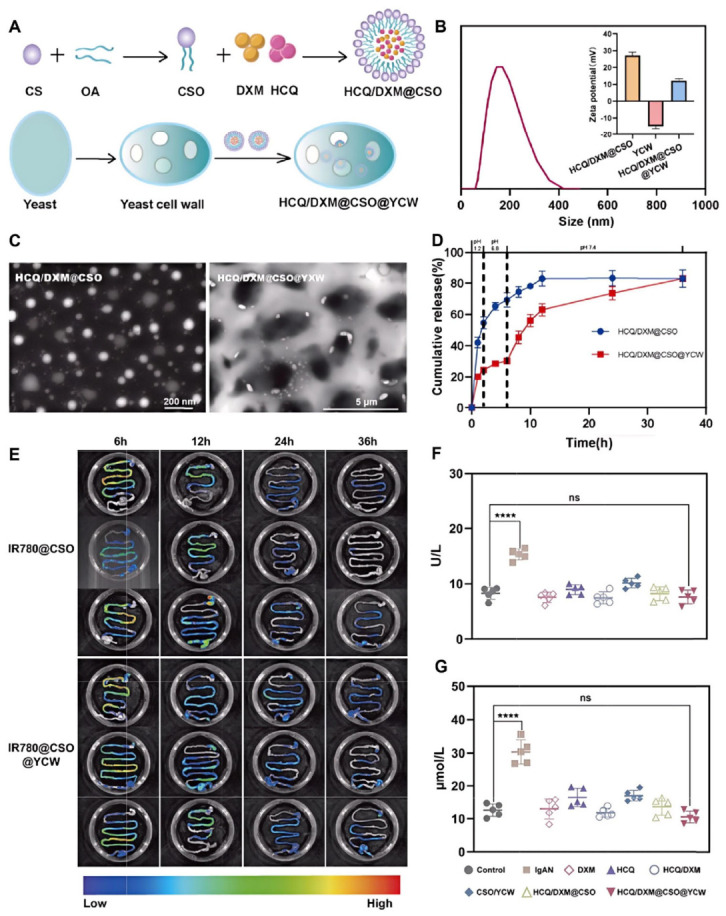
Targeting the gut-kidney axis with yeast cell wall (YCW) microcapsules for IgA nephropathy treatment. (**A**) Fabrication of the HCQ/DXM@CSO@YCW system, where drugs (HCQ/DXM) are encapsulated within YCWs to target intestinal lymphoid tissues. (**B**–**D**) The system shows uniform morphology and pH-responsive release characteristics. (**E**) In vivo biodistribution confirms the specific retention of YCW-coated particles in the intestine, facilitating mucosal immunity modulation. (**F**,**G**) Biochemical analysis confirms that the targeted delivery system significantly alleviates renal injury in IgAN mice, outperforming free drug administration. *n* = 5. ****: statistically significant (*p* < 0.0001); ns.: not significant (*p* > 0.05) [34]. Copyright © 2025 American Chemical Society.

Sun et al. engineered an oral pectin gel incorporating budesonide-encapsulated yeast microcapsules (NYPs@Gel) to regulate the gut-kidney axis for IgAN treatment [11]. This system leverages yeast microcapsules to shield budesonide, enhancing its accumulation in the GALT. Meanwhile, the pectin gel prolongs retention time and enables sustained release. NYPs@Gel significantly reduced inflammation and enteric IgA production, mitigated the systemic adverse effects of budesonide, restored gut microbiota balance, and marked ameliorated IgAN pathology. Similarly, Tian et al. developed a yeast cell wall (YCW)-coated, CSO carrier for the co-delivery of hydroxychloroquine (HCQ) and dexamethasone (DXM), designed for ileum-targeted delivery of treatment IgAN [34] (Figure 13A–D). This system demonstrated targeted uptake by dendritic cells and macrophages in vitro. In vivo, it significantly reduced serum IgA levels, renal IgA deposition, and inflammatory markers, while improving renal histology (Figure 13E–G).

Collectively, these studies indicate that YGPs hold significant potential as an oral, targeted delivery platform for the treatment of IgA nephropathy.

### 7.4. Vaccine Delivery

**Figure 14:**
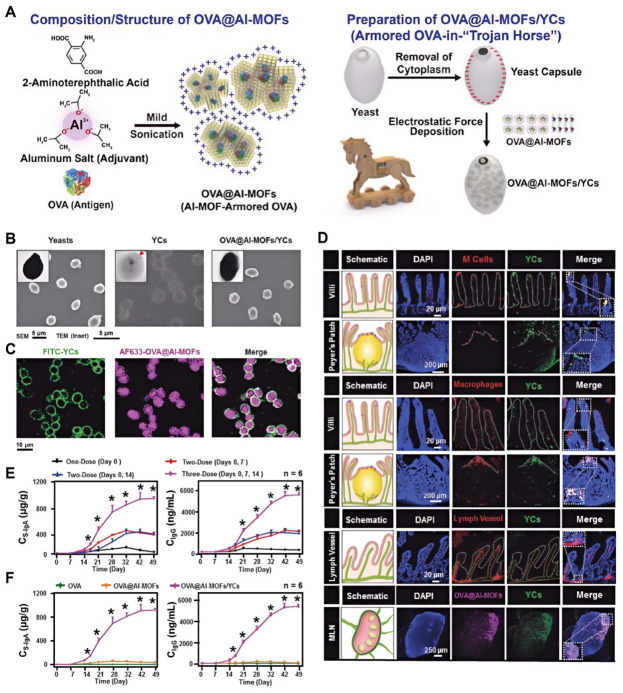
Yeast capsules (YCs) as a biomimetic “Trojan Horse” for oral vaccine delivery. (**A**) Fabrication of the core–shell hybrid system (OVA@Al-MOFs/YCs), integrating the adjuvant effect of Al-MOFs with the targeting capability of YCs. (**B**,**C**) Morphological verification confirms the integrity of the yeast shell after nanoparticle loading. (**D**) Mechanism of action: The YCs mediate effective transport across the intestinal epithelium via M cells in Peyer’s patches, facilitating antigen delivery to mesenteric lymph nodes (MLN). (**E**,**F**) The system induces potent and sustained systemic (IgG) and mucosal (IgA) immunity in mice, demonstrating its potential for oral vaccination. *: statistically significant (*p* < 0.05) [22]. Copyright © 2019 WILEY-VCH Verlag GmbH & Co. KGaA, Weinheim.

YGPs have garnered significant attention as oral vaccine delivery vehicles due to their inherent immunostimulatory properties and ability to target the gut immune system. Zhang et al. demonstrated that YGPs can act as “Trojan horses” to exploit the M cell transport pathway in Peyer’s patches. This mechanism enables the efficient delivery of antigen-adjuvant complexes (Al-MOFs) to lymphoid tissues, eliciting potent immune activation [30]. Similarly, Miao et al. developed a “Trojan Horse”-like transport platform utilizing yeast capsules (YCs) to carry an aluminum-based metal–organic framework (Al-MOF)-encapsulated antigen for oral vaccination, designed to overcome gastrointestinal and mucosal barriers (Figure 14A–C). This system demonstrated robust physical protection against degradative GI conditions in vitro, alongside targeted macrophage uptake and enhanced stimulation of immune activation markers. In vivo, it specifically targeted intestinal M cells for transepithelial transport and accumulated in mesenteric lymph nodes, significantly inducing potent and long-lasting mucosal S-IgA and systemic serum IgG immune responses (Figure 14D–F) [22]. Du et al. developed oral yeast-cell microcapsule (YCM) DNA vaccines targeting Clostridium perfringens type A [26]. Their results demonstrated that YCM-mediated vaccination induced effective immune responses in mice while simultaneously modulating gut microbiota and enhancing bacterial richness, presenting a promising strategy for preventing C. perfringens-induced diseases. Similarly, Hou et al. developed a sustained-release vaccine by loading tumor antigens and the TLR9 agonist CpG onto yeast β-glucan particles; this system induced potent, persistent immune responses that significantly suppressed tumor growth [28]. Miao et al. further reviewed the potential of yeast microcapsules and polymeric β-glucans as novel carriers targeting the gut lymphatic system for mRNA vaccine delivery [20].

Collectively, yeast microcapsules overcome the limitations of traditional delivery methods while effectively activating intestinal mucosal immunity, holding significant promise for the development of novel oral vaccines.

### 7.5. Treatment of Other Diseases

Beyond the primary applications discussed above, yeast microcapsules have demonstrated potential in treating a broader spectrum of diseases. Zhang et al. engineered self-propelled yeast micromotors (Cur@CaY-robots) encapsulating curcumin for gastritis therapy. These constructs effectively penetrated the dense gastric mucus layer, enhanced drug accumulation in gastric tissues, and restored gastric motility in murine models of gastritis [33]. Similarly, Jaecklein et al. developed yeast-derived glucan lipid particles (GLPs) for the targeted delivery of anti-tuberculosis drugs, demonstrating increased pulmonary drug retention and reduced bacterial load in Mycobacterium tuberculosis-infected mice [132]. Furthermore, Ray et al. highlighted the potential of orally targeted nanoparticles—including yeast microcapsules—for treating non-gastrointestinal conditions, such as neurodegenerative disorders, via entero-lymphatic transport mechanisms [2].

Collectively, these diverse applications underscore the versatility and efficacy of yeast microcapsules as oral drug delivery platforms, suggesting broad prospects for future clinical translation.

## 8. Conclusions and Perspectives

As a novel oral drug delivery carrier, YGPs have achieved breakthrough progress in the field of precision medicine in recent years, owing to their excellent biocompatibility, unique hollow and porous architecture, inherent immunomodulatory activity, and natural targeting capability toward intestinal immune cells. Although unmodified native YGPs face limitations such as premature drug leakage due to their porous nature and insufficient stimuli-responsiveness, the introduction of cutting-edge composite material engineering (e.g., chitosan and alginate coatings, metal–phenolic network assembly, and in situ mineralization of inorganic nanoparticles) has significantly enhanced their drug shielding efficacy, microenvironment-responsive intelligent release, targeting precision, and synergistic multi-drug delivery capabilities. These innovative functionalization strategies not only broaden the application of YGPs in complex disease models such as inflammatory bowel disease, malignant tumors, and IgA nephropathy but also demonstrate irreplaceable advantages in the field of oral vaccine delivery.

While current yeast-based delivery systems demonstrate remarkable targeted therapeutic efficacy, their successful clinical translation hinges heavily on the effective loading of drugs. The stability of the encapsulated substance during the loading process is a critical issue that must be addressed with caution. Traditional encapsulation methods often rely on high temperatures (e.g., spray drying) or concentrated organic solvents (e.g., ethanol or DMSO) to dissolve hydrophobic drugs and facilitate their diffusion across the robust yeast cell wall. However, these conditions pose a significant threat to the physicochemical stability of sensitive payloads. For instance, elevated temperatures can lead to the irreversible denaturation of proteins and peptides or the degradation of thermo-sensitive small molecules. Similarly, exposure to strong organic solvents can induce conformational changes, resulting in the loss of biological activity and raising concerns regarding residual solvent toxicity. Consequently, there is a paradigm shift in recent encapsulation developments toward mild, aqueous-based strategies. Techniques such as in situ biomineralization, metal–phenolic network coordination, and electrostatic self-assembly are highly favored, as they circumvent the need for high temperatures and organic solvents, thereby preserving the structural integrity and biological activity of the loaded substances while maximizing their therapeutic efficacy.

In addition, to effectively translate these academic achievements into clinical applications, future research must shift its focus from laboratory-scale fine synthesis to green, scalable, and industrialized standard manufacturing processes, thereby ensuring precise control over physicochemical properties and high batch-to-batch consistency. Concurrently, the design of YGP delivery platforms should move beyond single-environment responsiveness and evolve toward intelligent systems equipped with logic-gating mechanisms, enabling them to precisely navigate and dynamically adapt to complex pathological microenvironments characterized by tumor heterogeneity or inflammatory gradients.

Furthermore, comprehensively elucidating the in vivo pharmacokinetics and degradation kinetics of oral β-glucan systems is an essential prerequisite for clinical translation. Given the complexity of the intestinal microecology, the intricate interactions between YGPs and the gut microbiota—particularly whether they can sustainably promote the production of beneficial metabolites like SCFAs, or remotely regulate distal physiological processes via the gut–brain and gut–lung axes—urgently require more systematic metagenomic and metabolomic investigations.

Considering these profound biological effects, conducting rigorous long-term safety and immune tolerance evaluations in large animal models is particularly crucial, not only to screen for potential cumulative toxicity but also to ensure that composite materials undergoing complex chemical modifications do not induce unexpected immune overactivation in vivo.

Accompanied by the deep interdisciplinary convergence of materials science, basic immunology, and biomedicine, highly integrated and intelligent YGPs composite delivery systems are poised to become the core cornerstone driving the development of next-generation oral precision drug delivery.

## Figures and Tables

**Table 1:** Summary of Functionalization and Optimization Strategies for YGPs.

Strategy	Materials		Loaded Drugs	Role of Materials	Diseases of Application	References
Nanoparticle Encapsulation Strategy	Mesoporous polydopamine nanoparticles (MPDA)		Diallyl trisulfide (H_2_S prodrug); Manganese dioxide (MnO_2_) nanozymes	Highly efficient loading (49.2%) and stable encapsulation of the H_2_S prodrug DATS via π-π stacking interactions	UC	[103]
	Supramolecular nano-delivery system (Man-CUR NPs)		Curcumin (Cur)	pH/ROS dual-responsive drug release; Macrophage-Targeted Drug Delivery	UC	[16]
	Disulfide-crosslinked HA-rhein nanoparticles (HA-Cys-RH)		Emodin (Emo)	Enhanced drug stability and encapsulation efficiency; Macrophage-Targeted Drug Delivery	UC	[104]
	Hyaluronic acid (HA) and polyethylenimine (PEI) modified ovalbumin NPs (HA/PEI-RH NPs)		Rhein (RH)	Enhanced drug stability and encapsulation efficiency; Macrophage-Targeted Drug Delivery	UC	[17]
	Humic acid-iron complexed nanoparticles (HA-Fe NPs)		/	Efficient adsorption of aflatoxin B_1_ (AFB_1_)	Hepatic and renal injury	[105]
	Oleic acid-grafted chitosan (CSO)		Hydroxychloroquine (HCQ) and dexamethasone (DXM)	Enhanced drug stability and encapsulation efficiency	IgAN	[34]
Metal–Phenolic Network Assembly Strategy	Metal–polyphenol networks (MPNs) formed by the self-assembly of epigallocatechin gallate (EGCG) and Fe^3+^		Curcumin (Cur)	Enhanced drug encapsulation efficiency; Antioxidant activity	UC	[15]
	Metal–polyphenol networks (MPNs) formed by the self-assembly of epigallocatechin gallate (EGCG) and Mn^2+^		Berberine (BBR)	Enhanced drug encapsulation efficiency; Antioxidant activity	UC	[52]
	Metal–polyphenol networks (MPNs) formed by the self-assembly of epigallocatechin gallate (EGCG) and Fe^3+^		Nobiletin (NOB)	Enhanced drug encapsulation efficiency; Antioxidant activity	UC	[14]
	Metal–polyphenol networks (MPNs) formed by the self-assembly of dihydromyricetin (DHM) and Zn^2+^		/	Anti-inflammatory and antioxidant activity	UC	[25]
In Situ Biomineralization Strategy	Induce inorganic/polymeric mineralization or high hydrophobicity within the cavity to physically immobilize loaded drugs.	CaCO_3_ mineralized skeleton	Curcumin (Cur)	Enhanced drug encapsulation efficiency	Gastritis	[33]
		Tetraethylorthosilicate (TEOS)	Bovine serum albumin (BSA) as model protein	Enhanced protein thermal stability	Oral delivery of proteins	[65]
		/	Avenanthramide-C (AVN-C)	High hydrophobicity-mediated enhancement of AVN-C stability and sustained release	/	[57]
		3-O-desacyl-4′-monophosphoryl lipid A (a TLR4 agonist) and aluminum salts	The total extract (TE) of *T. gondii* lysate	Highly efficient antigen loading; Enhanced cellular immunity	*Toxoplasma gondii* (*T. gondii*) infection	[106]
		Aluminum salt (Alum)	Hepatitis B surface antigen (HBsAg)	Prolonged in vivo antigen retention; enhanced humoral immune response	Oral delivery of vaccines	[107]
Surface Chemical Modification and External Gel Encapsulation Strategy	Drug encapsulation and controlled release are achieved by grafting or multilayer coating compounds onto YGP surfaces.	HA	Doxorubicin (DOX)	Cancer cell-targeted drug delivery	Breast cancer	[71]
		Positively charged polyethyleneimine	Antigen	Enhanced antigen loading efficiency	Oral delivery of vaccines	[108]
		Positively charged chitosan and negatively charged chondroitin sulfate	Anthocyanins	Enhanced drug encapsulation efficiency and retention rate	/	[109]
		Chitosan	Cefadroxil	Enhanced drug encapsulation efficiency and retention rate	UC	[35]
		Alginate (ALG) and CaCl_2_	Insulin	Enhanced drug encapsulation efficiency and retention rate	Diabetes Mellitus (MD)	[110]
		Chitosan/sodium alginate (CS/SA)	Emodin (EMO), asiatic acid (AA) and *Lactobacillus casei* Zhang (*L. casei* Zhang)	Enhanced drug encapsulation efficiency and retention rate	Renal fibrosis	[111]

## Data Availability

No new data were created or analyzed in this study.

## Abbreviations

Abbreviation	Meaning
YGPs	yeast glucan particles
GALT	gut-associated lymphoid tissue
DSS	drug delivery systems
IBD	inflammatory bowel disease
GI	gastrointestinal
GRAS	Generally Recognized As Safe
PRRs	pattern recognition receptors
APCs	antigen-presenting cells
GPI	glycosylphosphatidylinositol
LC	loading capacity
EE	encapsulation efficiency
TEM	transmission electron microscope
QDs	Quantum Dots
FITC	fluorescein isothiocyanate
NOB	nobiletin
YM	yeast microcapsule
MPN	metal polyphenol network
AVN-C	avenanthramide-c
DOX	doxorubicin
NPs	Nanoparticles
CLSM	confocal laser scanning microscopy
PPs	Peyer’s patches
PAMP	pathogen-associated molecular pattern
CR3	complement receptor 3
DCs	dendritic cells
ROS	reactive oxygen species
SCFAs	Short-Chain Fatty Acids
MPDA	mesoporous polydopamine
MnO_2_	manganese dioxide
DATS	diallyl trisulfide
H_2_S	hydrogen sulfide
Lf	lactoferrin
SEM	Scanning Electron Microscopy
OVA	ovalbumin
PEI	polyethyleneimine
HA	hyaluronic acid
AFB1	aflatoxin B1
TA	tannic acid
EGCG	epigallocatechin gallate
Cur	curcumin
DHM	dihydromyricetin
CaCO_3_	calcium carbonate
TEOS	tetraethyl orthosilicate
PEG	polyethylene glycol
LbL	Layer-by-Layer
CS	chitosan
CSO	oleic acid-modified chitosan
UC	ulcerative colitis
YPs	yeast particles
OA	osteoarthritis
RA	rheumatoid arthritis
PTOA	post-traumatic osteoarthritis
YMPs	yeast microcapsules
AS	atherosclerosis
TAMs	tumor-associated macrophages
TME	tumor microenvironment
IgAN	IgA Nephropathy
YCW	yeast cell wall
HCQ	hydroxychloroquine
YCs	yeast capsules
Al-MOF	aluminum-based metal–organic framework
GLPs	glucan lipid particles

## References

[1] Wu Y., Li P., Jiang Z., Sun X., He H., Yan P., Xu Y., Liu Y. (2023). Bioinspired Yeast-Based β-Glucan System for Oral Drug Delivery. Carbohydr. Polym..

[2] Ray S., Moonshi S.S., Ta H.T. (2025). “Therapies Through Gut:” Targeted Drug Delivery for Non-Gastrointestinal Diseases by Oral Administration. Adv. Healthc. Mater..

[3] Jin Y.-B., Wei Y.-S., Yang Y.-X., Feng K., Wu H. (2025). Improving the Colonic Release and Serum Glucose Regulation of Insulin by Co-Loaded a Prebiotic in Alginate Composite Microcapsules. Int. J. Biol. Macromol..

[4] Ejazi S.A., Louisthelmy R., Maisel K. (2023). Mechanisms of Nanoparticle Transport across Intestinal Tissue: An Oral Delivery Perspective. ACS Nano.

[5] Ensign L.M., Cone R., Hanes J. (2012). Oral Drug Delivery with Polymeric Nanoparticles: The Gastrointestinal Mucus Barriers. Adv. Drug Deliv. Rev..

[6] Subramanian D.A., Langer R., Traverso G. (2022). Mucus Interaction to Improve Gastrointestinal Retention and Pharmacokinetics of Orally Administered Nano-Drug Delivery Systems. J. Nanobiotechnol..

[7] Šalamúnová P., Saloň I., Ruphuy G., Kroupová J., Balouch M., Hanuš J., Štěpánek F. (2021). Evaluation of β-Glucan Particles as Dual-Function Carriers for Poorly Soluble Drugs. Eur. J. Pharm. Biopharm..

[8] Fu D., Fu J., Li J., Tang Y., Shao Z., Zhou D., Song L. (2022). Efficient Encapsulation of Curcumin into Spent Brewer’s Yeast Using a pH-Driven Method. Food Chem..

[9] Rotrekl D., Šalamúnová P., Paráková L., Baďo O., Saloň I., Štěpánek F., Hanuš J., Hošek J. (2021). Composites of Yeast Glucan Particles and Curcumin Lead to Improvement of Dextran Sulfate Sodium-Induced Acute Bowel Inflammation in Rats. Carbohydr. Polym..

[10] Huang D., Zou M., Xu C., Wang Y., Xu Z., Zhang W., Tang S., Weng Z. (2024). Colon-Targeted Oral Delivery of Hydroxyethyl Starch–Curcumin Microcapsules Loaded with Multiple Drugs Alleviates DSS-Induced Ulcerative Colitis in Mice. Macromol. Biosci..

[11] Sun A., Luo Q., Liu H., Yang W., Liu J., Shi X., Nie Y., Sun J., Sun M., Liu L. (2025). An Orally Budesonide-Loaded Yeast Microcapsules-Based Gel Relieves IgA Nephropathy via the Modulation of Gut-Kidney Axis. Nano Today.

[12] Kang Y., She Y., Zang Y., Yuan M., Niu G., Tian X., Zhang L., Lin J., Yang M., Pei Z. (2025). Biohybrid Microrobot Enteric-Coated Microcapsule for Oral Treatment of Colorectal Cancer. Adv. Mater..

[13] Li L., Dai L., Lin M., He S., Du H., Lin D., Wang Y., Zhang F., Tao S., Sun X. (2025). Colonic Submucosa Targeted Delivery of Probiotic and Rhein for Ulcerative Colitis Treatment. Adv. Sci..

[14] Yang J., Xia X., Du M., Cheng S., Zhu B., Xu X. (2024). Highly Effective Nobiletin–MPN in Yeast Microcapsules for Targeted Modulation of Oxidative Stress, NLRP3 Inflammasome Activation, and Immune Responses in Ulcerative Colitis. J. Agric. Food Chem..

[15] Li J., Song J., Deng Z., Yang J., Wang X., Gao B., Zhu Y., Yang M., Long D., Luo X. (2024). Robust Reactive Oxygen Species Modulator Hitchhiking Yeast Microcapsules for Colitis Alleviation by Trilogically Intestinal Microenvironment Renovation. Bioact. Mater..

[16] Han X., Luo R., Qi S., Wang Y., Dai L., Nie W., Lin M., He H., Ye N., Fu C. (2023). “Dual Sensitive Supramolecular Curcumin Nanoparticles” in “Advanced Yeast Particles” Mediate Macrophage Reprogramming, ROS Scavenging and Inflammation Resolution for Ulcerative Colitis Treatment. J. Nanobiotechnol..

[17] Chen Q., Luo R., Han X., Zhang J., He Y., Qi S., Pu X., Nie W., Dong L., Xu H. (2021). Entrapment of Macrophage-Target Nanoparticles by Yeast Microparticles for Rhein Delivery in Ulcerative Colitis Treatment. Biomacromolecules.

[18] Yang F., Shang S., Qi M., Xiang Y., Wang L., Wang X., Lin T., Hao D., Chen J., Liu J. (2023). Yeast Glucan Particles: An Express Train for Oral Targeted Drug Delivery Systems. Int. J. Biol. Macromol..

[19] Bastos R., Oliveira P.G., Gaspar V.M., Mano J.F., Coimbra M.A., Coelho E. (2022). Brewer’s Yeast Polysaccharides—A Review of Their Exquisite Structural Features and Biomedical Applications. Carbohydr. Polym..

[20] Miao Y., Lin Y., Chen K., Luo P., Chuang S., Yu Y., Tai H., Chen C., Lin K., Sung H. (2021). Engineering Nano- and Microparticles as Oral Delivery Vehicles to Promote Intestinal Lymphatic Drug Transport. Adv. Mater..

[21] He L., Zhu Z., Qi C. (2024). β-Glucan—A Promising Immunocyte-Targeting Drug Delivery Vehicle: Superiority, Applications and Future Prospects. Carbohydr. Polym..

[22] Miao Y., Pan W., Chen K., Wei H., Mi F., Lu M., Chang Y., Sung H. (2019). Engineering a Nanoscale Al-MOF-Armored Antigen Carried by a “Trojan Horse”-Like Platform for Oral Vaccination to Induce Potent and Long-Lasting Immunity. Adv. Funct. Mater..

[23] Sarkar N., Mahajan A.A., Pathak S., Seth P., Chowdhury A., Ghose I., Das S., Chowdhury R., Bera A., Dey A. (2025). Beta-Glucans in Biotechnology: A Holistic Review with a Special Focus on Yeast. Bioengineering.

[24] Gohri J., Mir M.B., Rohilla S., Sharma M., Mir M.B., Mir S.A. (2025). Beta-Glucan Based Bioactive Delivery Systems. Beta-Glucan: Sources, Properties and Applications.

[25] Chai M., Zhu Y., Chen L., Zhang S., Huang Y., Zhang M., Jin W. (2025). Yeast Microcapsules Encapsulating Metal-Phenolic Nanozymes Alleviate Ulcerative Colitis by Mitigating Oxidative Stress and Modulating the Gut Microbiota. Mater. Today Bio.

[26] Du L., Jia S., Zhang W., Cai C., Liu Y., Wang C., Zhu Y., Ma X., Yang X., Wei Z. (2024). Oral Yeast-Cell Microcapsule-Mediated DNA Vaccines Against Clostridium Perfringens Induce Effective Intestinal Immunity and Modulate Gut Microbiota. Vaccines.

[27] Choi W., Shin W.-R., Kim Y.-H., Min J. (2023). Inducing a Proinflammatory Response with Bioengineered Yeast Vacuoles with TLR2-Binding Peptides (Vac^T2BP^) as a Drug Carrier for Daunorubicin Delivery. ACS Appl. Mater. Interfaces.

[28] Hou Y., Liu R., Hong X., Zhang Y., Bai S., Luo X., Zhang Y., Gong T., Zhang Z., Sun X. (2021). Engineering a Sustained Release Vaccine with a Pathogen-Mimicking Manner for Robust and Durable Immune Responses. J. Control. Release.

[29] Silva de Macêdo L., Sousa de Pinho S., Duarte Silva A.J., De Moura I.A., Flores Espinoza B.C., Viana da Invenção M.C., Silva Novis P.V., Turiah Machado da Gama M.A., Do Nascimento Carvalho M., Rosa Sales Leal L. (2024). Understanding Yeast Shells: Structure, Properties and Applications. ADMET DMPK.

[30] Zhang R., Qin X., Lu J., Xu H., Zhao S., Li X., Yang C., Kong L., Guo Y., Zhang Z. (2023). Chemodynamic/Photothermal Synergistic Cancer Immunotherapy Based on Yeast Microcapsule-Derived Au/Pt Nanoparticles. ACS Appl. Mater. Interfaces.

[31] Kiran N.S., Subramaniam D., Yashaswini C., Chatterjee A., Prajapati B., Alsaidan O.A., Alzarea S.I., Bhattacharya S. (2026). Advancing β-Glucan-Based Immunomodulation and Nanotherapeutic Strategies for Cancer Biotherapy. Cancer Biother. Radiopharm..

[32] Zhang B., Pan H., Chen Z., Yin T., Zheng M., Cai L. (2023). Twin-Bioengine Self-Adaptive Micro/Nanorobots Using Enzyme Actuation and Macrophage Relay for Gastrointestinal Inflammation Therapy. Sci. Adv..

[33] Zhang L., Zhang B., Liang R., Ran H., Zhu D., Ren J., Liu L., Ma A., Cai L. (2023). A Dual-Biomineralized Yeast Micro-/Nanorobot with Self-Driving Penetration for Gastritis Therapy and Motility Recovery. ACS Nano.

[34] Tian C., Yan M., Guo J., Zhou Y., Du B., Cheng G. (2025). Yeast Cell Wall-Mediated Ileal Targeted Delivery System for IgA Nepharopathy Therapy. ACS Biomater. Sci. Eng..

[35] Mohammed A.I.S.G., Hmingthansanga V., Das P., Arumugam S., Rajdev B., Naidu V.G.M., Ravichandiran V., Natesan S. (2026). Chitosan Coated Yeast Microcapsules for Efficient Delivery of Cefadroxil for the Treatment of Inflammatory Bowel Disease. Int. J. Biol. Macromol..

[36] Meligi N.M., Dyab A.K.F. (2023). Natural Sporopollenin Microcapsules: Biological Evaluation and Application in Regulating Hepatic Toxicity of Diclofenac Sodium In Vivo. Biomater. Sci..

[37] Gow N.A.R., Lenardon M.D. (2023). Architecture of the Dynamic Fungal Cell Wall. Nat. Rev. Microbiol..

[38] Kang X., Kirui A., Muszyński A., Widanage M.C.D., Chen A., Azadi P., Wang P., Mentink-Vigier F., Wang T. (2018). Molecular Architecture of Fungal Cell Walls Revealed by Solid-State NMR. Nat. Commun..

[39] Yang F., Shang S., Qi M., Hou M., Xiang Y., Shen J., Tong Y., Zhang Y., Liu J., Wu Q. (2024). Yeast-Originated Drug Carriers: Comprehensive Study from Preparation to Functional Evaluation. J. Drug Deliv. Sci. Technol..

[40] Klis F.M., Mol P., Hellingwerf K., Brul S. (2002). Dynamics of Cell Wall Structure in *Saccharomyces cerevisiae*. FEMS Microbiol. Rev..

[41] Lin H., Han R., Wu W. (2024). Glucans and Applications in Drug Delivery. Carbohydr. Polym..

[42] Mahmoud Amer E., Saber S.H., Abo Markeb A., Elkhawaga A.A., Mekhemer I.M.A., Zohri A.-N.A., Abujamel T.S., Harakeh S., Abd-Allah E.A. (2021). Enhancement of β-Glucan Biological Activity Using a Modified Acid-Base Extraction Method from Saccharomyces Cerevisiae. Molecules.

[43] Saloň I., Hanuš J., Ulbrich P., Štěpánek F. (2016). Suspension Stability and Diffusion Properties of Yeast Glucan Microparticles. Food Bioprod. Process..

[44] Yan M., Liu G., Liu S., Liu J., Li H., Wang H., Zou Y., Pan C., Zhou F., Zeng X. (2025). Ultrasonic-Assisted Enzymatic Extraction, Physicochemical Properties and Prebiotic Activities of Polysaccharides from Saccharomyces Cerevisiae Spore Wall. Ultrason. Sonochem..

[45] Chemat F., Rombaut N., Sicaire A.-G., Meullemiestre A., Fabiano-Tixier A.-S., Abert-Vian M. (2017). Ultrasound Assisted Extraction of Food and Natural Products. Mechanisms, Techniques, Combinations, Protocols and Applications. A Review. Ultrason. Sonochem..

[46] Yuan H., He Y., Zhang H., Ma X. (2022). Ultrasound-Assisted Enzymatic Hydrolysis of Yeast β-Glucan Catalyzed by β-Glucanase: Chemical and Microstructural Analysis. Ultrason. Sonochem..

[47] Ma X., Dong L., He Y., Chen S. (2022). Effects of Ultrasound-Assisted H2O2 on the Solubilization and Antioxidant Activity of Yeast β-Glucan. Ultrason. Sonochem..

[48] Varelas V., Liouni M., Calokerinos A.C., Nerantzis E.T. (2016). An Evaluation Study of Different Methods for the Production of *β*-D-glucan from Yeast Biomass. Drug Test. Anal..

[49] Borchani C., Fonteyn F., Jamin G., Paquot M., Blecker C., Thonart P. (2014). Enzymatic Process for the Fractionation of Baker’s Yeast Cell Wall (*Saccharomyces cerevisiae*). Food Chem..

[50] Ren T., Gou J., Sun W., Tao X., Tan X., Wang P., Zhang Y., He H., Yin T., Tang X. (2018). Entrapping of Nanoparticles in Yeast Cell Wall Microparticles for Macrophage-Targeted Oral Delivery of Cabazitaxel. Mol. Pharm..

[51] Rajabi A., Nejati M., Homayoonfal M., Arj A., Razavi Z.S., Ostadian A., Mohammadzadeh B., Vosough M., Karimi M., Rahimian N. (2024). Doxorubicin-Loaded Zymosan Nanoparticles: Synergistic Cytotoxicity and Modulation of Apoptosis and Wnt/β-Catenin Signaling Pathway in C26 Colorectal Cancer Cells. Int. J. Biol. Macromol..

[52] Feng X., Xie Q., Xu H., Zhang T., Li X., Tian Y., Lan H., Kong L., Zhang Z. (2022). Yeast Microcapsule Mediated Natural Products Delivery for Treating Ulcerative Colitis through Anti-Inflammatory and Regulation of Macrophage Polarization. ACS Appl. Mater. Interfaces.

[53] Soto E.R., Rus F., Mirza Z., Ostroff G.R. (2023). Yeast Particles for Encapsulation of Terpenes and Essential Oils. Molecules.

[54] Soto E.R., Rus F., Li H., Garceau C., Chicca J., Elfawal M., Gazzola D., Nielsen M.K., Urban J.F., Aroian R.V. (2021). Yeast Particle Encapsulation of Scaffolded Terpene Compounds for Controlled Terpene Release. Foods.

[55] Pinho S.S.D., Invenção M.D.C.V., Silva A.J.D., Macêdo L.S.D., Espinoza B.C.F., Leal L.R.S., Da Gama M.A.T.M., Moura I.A.D., Silva M.E.D.S., Souza D.V.S.D. (2024). Pichia Pastoris-Derived β-Glucan Capsules as a Delivery System for DNA Vaccines. Vaccines.

[56] Dadkhodazade E., Mohammadi A., Shojaee-Aliabadi S., Mortazavian A.M., Mirmoghtadaie L., Hosseini S.M. (2018). Yeast Cell Microcapsules as a Novel Carrier for Cholecalciferol Encapsulation: Development, Characterization and Release Properties. Food Biophys..

[57] He L., Zhu Y., Shen X., Chen G., Xiao H., Wang J., Tan C. (2024). Yeast Cell Wall Capsules for Delivery of Oat Biomarker Avenanthramide-C. Food Chem..

[58] Bao X., Rong S., Fu Q., Liu H., Han Y., Liu F., Ye Z., Chen S. (2023). Zein-Yeast Carboxymethyl Glucan Particles Formed by Anti-Solvent Precipitation for Encapsulating Resveratrol. Int. J. Biol. Macromol..

[59] Ismail A.S., Sreedharan D.K., Ng Z.J., Tan J.S. (2025). Microencapsulation of Lactobacillus Cells Utilizing the β-Glucan-Rich Cell Wall of Saccharomyces Cerevisiae for Enhanced Stability and Efficacy. Int. J. Biol. Macromol..

[60] Wu Y., Wang X., Yin Z., Dong J. (2023). Geotrichum Candidum Arthrospore Cell Wall Particles as a Novel Carrier for Curcumin Encapsulation. Food Chem..

[61] Young S., Dea S., Nitin N. (2017). Vacuum Facilitated Infusion of Bioactives into Yeast Microcarriers: Evaluation of a Novel Encapsulation Approach. Food Res. Int..

[62] Ren T., Zheng X., Bai R., Yang Y., Jian L. (2021). Utilization of PLGA Nanoparticles in Yeast Cell Wall Particle System for Oral Targeted Delivery of Exenatide to Improve Its Hypoglycemic Efficacy. Int. J. Pharm..

[63] Luo Y., Wang Q. (2014). Recent Development of Chitosan-Based Polyelectrolyte Complexes with Natural Polysaccharides for Drug Delivery. Int. J. Biol. Macromol..

[64] Rajaei A., Salarbashi D., Tafaghodi M., Sabeti Z., Sabbagh F., Rakhshani S., Kamali H., Fahmideh-Rad E. (2023). Evaluation of Antimicrobial and Structural Properties of Thyme Essential Oil-Loaded Chitosan-Capric Acid and Chitosan-Stearic Acid Nanogels. J. Food Qual. Hazards Control.

[65] Soto E.R., Specht C.A., Rus F., Lee C.K., Abraham A., Levitz S.M., Ostroff G.R. (2023). An Efficient (Nano) Silica—In Glucan Particles Protein Encapsulation Approach for Improved Thermal Stability. J. Control. Release.

[66] Gümüşay Ö.A., Cerit İ., Demirkol O. (2025). Utilization of Yeast Cells as Alternative Carriers in the Microencapsulation of Black Chokeberry (*Aronia melanocarpa*) Phenolic Extract. Foods.

[67] Semouma D., Laib I., Laib D.E., Chenchouni H., Rahmani Y., Fekrache F., Hadef A., Bensouici C., Barkat M. (2024). Microencapsulation of Myrtus Communis Extracts in Saccharomyces Cerevisiae Cells: Effects on Phenolic Content and Antioxidant Capacity, Physical Characterization and Molecular Docking Analysis. Food Bioprocess Technol..

[68] Karaman K. (2023). Encapsulation and Characterization of Catechin and Epicatechin Microcapsules Using Yeast Cell Biocarriers. Eur. Food Sci. Eng..

[69] Zhou X., Zhang X., Han S., Dou Y., Liu M., Zhang L., Guo J., Shi Q., Gong G., Wang R. (2017). Yeast Microcapsule-Mediated Targeted Delivery of Diverse Nanoparticles for Imaging and Therapy via the Oral Route. Nano Lett..

[70] Young S., Rai R., Nitin N. (2020). Bioaccessibility of Curcumin Encapsulated in Yeast Cells and Yeast Cell Wall Particles. Food Chem..

[71] He F., Xie C., Xu X. (2023). Hyaluronic Acid-Modified Yeast β-Glucan Particles Delivering Doxorubicin for Treatment of Breast Cancer. Carbohydr. Polym..

[72] Zhang Z., Lu Y., Qi J., Wu W. (2021). An Update on Oral Drug Delivery via Intestinal Lymphatic Transport. Acta Pharm. Sin. B.

[73] Murphy E.J., Rezoagli E., Collins C., Saha S.K., Major I., Murray P. (2023). Sustainable Production and Pharmaceutical Applications of β-Glucan from Microbial Sources. Microbiol. Res..

[74] Miao Y., Chen K., Chen C., Mi F., Lin Y., Chang Y., Chiang C., Wang J., Lin K., Sung H. (2021). A Noninvasive Gut-to-Brain Oral Drug Delivery System for Treating Brain Tumors. Adv. Mater..

[75] Mata-Martínez P., Bergón-Gutiérrez M., del Fresno C. (2022). Dectin-1 Signaling Update: New Perspectives for Trained Immunity. Front. Immunol..

[76] De Marco Castro E., Calder P.C., Roche H.M. (2021). Β-1,3/1,6-Glucans and Immunity: State of the Art and Future Directions. Mol. Nutr. Food Res..

[77] Hu S., Meng Y., Guo L., Xu X. (2024). A Novel Strategy to Enhance Inhibition of Hela Cervical Cancer by Combining Lentinus β-Glucan and Autophagic Flux Blockage. Int. J. Biol. Macromol..

[78] Goodridge H.S., Reyes C.N., Becker C.A., Katsumoto T.R., Ma J., Wolf A.J., Bose N., Chan A.S.H., Magee A.S., Danielson M.E. (2011). Activation of the Innate Immune Receptor Dectin-1 upon Formation of a ‘Phagocytic Synapse’. Nature.

[79] Li M., Yu Y. (2021). Innate Immune Receptor Clustering and Its Role in Immune Regulation. J. Cell Sci..

[80] Geller A., Shrestha R., Yan J. (2019). Yeast-Derived β-Glucan in Cancer: Novel Uses of a Traditional Therapeutic. Int. J. Mol. Sci..

[81] Zhong X., Wang G., Li F., Fang S., Zhou S., Ishiwata A., Tonevitsky A.G., Shkurnikov M., Cai H., Ding F. (2023). Immunomodulatory Effect and Biological Significance of β-Glucans. Pharmaceutics.

[82] Volman J.J., Ramakers J.D., Plat J. (2008). Dietary Modulation of Immune Function by β-Glucans. Physiol. Behav..

[83] Quintin J., Saeed S., Martens J.H.A., Giamarellos-Bourboulis E.J., Ifrim D.C., Logie C., Jacobs L., Jansen T., Kullberg B.-J., Wijmenga C. (2012). Candida Albicans Infection Affords Protection against Reinfection via Functional Reprogramming of Monocytes. Cell Host Microbe.

[84] Brown G.D. (2006). Dectin-1: A Signalling Non-TLR Pattern-Recognition Receptor. Nat. Rev. Immunol..

[85] Netea M.G., Joosten L.A.B., Latz E., Mills K.H.G., Natoli G., Stunnenberg H.G., O’Neill L.A.J., Xavier R.J. (2016). Trained Immunity: A Program of Innate Immune Memory in Health and Disease. Science.

[86] Li M., Vultorius C., Bethi M., Yu Y. (2022). Spatial Organization of Dectin-1 and TLR2 during Synergistic Crosstalk Revealed by Super-Resolution Imaging. J. Phys. Chem. B.

[87] Rainer H., Goretzki A., Lin Y.-J., Schiller H.R., Krause M., Döring S., Strecker D., Junker A.-C., Wolfheimer S., Toda M. (2024). Characterization of the Immune-Modulating Properties of Different β-Glucans on Myeloid Dendritic Cells. Int. J. Mol. Sci..

[88] Thornton B.P., Vĕtvicka V., Pitman M., Goldman R.C., Ross G.D. (1996). Analysis of the Sugar Specificity and Molecular Location of the Beta-Glucan-Binding Lectin Site of Complement Receptor Type 3 (CD11b/CD18). J. Immunol..

[89] Manabe N., Yamaguchi Y. (2021). 3D Structural Insights into β-Glucans and Their Binding Proteins. Int. J. Mol. Sci..

[90] O’Brien X.M., Heflin K.E., Lavigne L.M., Yu K., Kim M., Salomon A.R., Reichner J.S. (2012). Lectin Site Ligation of CR3 Induces Conformational Changes and Signaling. J. Biol. Chem..

[91] McFarlin B.K., Venable A.S., Carpenter K.C., Henning A.L., Ogenstad S. (2017). Oral Supplementation with Baker’s Yeast Beta Glucan Is Associated with Altered Monocytes, T Cells and Cytokines Following a Bout of Strenuous Exercise. Front. Physiol..

[92] Cheong K.-L., Sabir A., Veeraperumal S., Quero F., Li R., Zhao Q., Tan K., Zhong S., Veerabagu U. (2025). Possibility of Alleviating Dextran Sulfate Sodium-Induced Colitis in Mice by Modulate Intestinal Barrier Function and Gut Microbiota with Laminarin Acetyl Esters. Carbohydr. Polym. Technol. Appl..

[93] Ma L., Lin X., Liang M., Long J., Qu X., Yu Y., Zhou Y., Cheng H. (2025). Effect of Grifola Frondosa Polysaccharide on Immune Function and Gut Microbiota in Mice. Acupunct. Herb. Med..

[94] Qu X., Ji Y., Long J., Zheng D., Qiao Z., Lin Y., Lu C., Zhou Y., Cheng H. (2025). Immuno-and Gut Microbiota-Modulatory Activities of β-1,6-Glucans from Lentinus Edodes. Food Chem..

[95] Kirmaz C., Bayrak P., Yilmaz O., Yuksel H. (2005). Effects of Glucan Treatment on the Th1/Th2 Balance in Patients with Allergic Rhinitis: A Double-Blind Placebo-Controlled Study. J. Investig. Allergol. Clin. Immunol..

[96] Briskey D., Pellow J., Beatson G., Tremblay A., Rao A., Tompkins T.A. (2025). Efficacy and Safety of a Particulate Yeast β-Glucan Preparation in the Treatment of Seasonal Allergic Rhinitis (BETALL): A Randomized Placebo-Controlled Crossover Trial Protocol. BMC Trials.

[97] Richter J., Svozil V., Král V., Dobiášová L.R., Vetvicka V. (2015). β-Glucan Affects Mucosal Immunity in Children with Chronic Respiratory Problems under Physical Stress: Clinical Trials. Ann. Transl. Med..

[98] Gaullier J.-M., Sleboda J., Ofjord E.S., Ulvestad E., Nurminiemi M., Moe C., Albrektsen T., Gudmundsen O. (2011). Supplementation with a Soluble Beta-Glucan Exported from Shiitake Medicinal Mushroom, *Lentinus edodes* (Berk.) Singer Mycelium: A Crossover, Placebo-Controlled Study in Healthy Elderly. Int. J. Med. Mushr..

[99] Vlassopoulou M., Yannakoulia M., Pletsa V., Zervakis G.I., Kyriacou A. (2021). Effects of Fungal Beta-Glucans on Health—A Systematic Review of Randomized Controlled Trials. Food Funct..

[100] Guo Y., Liu Y., Chen K., Cai L., Huang S., Zhang Y. (2024). Super Gastro-Resistant Microcapsules Based on CaCO_3_ Nanocrystal Buffered Alginate/Pectin Composites for Colon-Targeted Probiotic Delivery: In Vitro and in Vivo Evaluation. Adv. Compos. Hybrid. Mater..

[101] Bhagat S., Singh S. (2024). Nanominerals Packaged in pH-Responsive Alginate Microcapsules Exhibit Selective Delivery in Small Intestine and Enhanced Absorption and Bioavailability. Adv. Funct. Mater..

[102] Araújo D., Alves V.D., Lima S.A.C., Reis S., Freitas F., Reis M.A.M. (2020). Novel Hydrogels Based on Yeast Chitin-Glucan Complex: Characterization and Safety Assessment. Int. J. Biol. Macromol..

[103] Zhang X., Yang H., He Y., Zhang D., Lu G., Ren M., Lyu Y., Yuan Z., He S. (2025). Yeast-Inspired Orally-Administered Nanocomposite Scavenges Oxidative Stress and Restores Gut Immune Homeostasis for Inflammatory Bowel Disease Treatment. ACS Nano.

[104] Wang Y., Zhong S., Yang K., Luo R., Dai L., Zhong W., Ye Y., Fu C., Lin D., Li N. (2024). β-1,3-d-Glucan Particles-Based “Nest” Protected Co-Loaded Rhein and Emodin Regulates Microbiota and Intestinal Immunity for Ulcerative Colitis Treatment. Int. J. Biol. Macromol..

[105] Hamza Z.K., Hathout A.S., Ostroff G., Soto E., Sabry B.A., El-Hashash M.A., Hassan N.S., Aly S.E. (2022). Assessment of the Protective Effect of Yeast Cell Wall Β-glucan Encapsulating Humic Acid Nanoparticles as an Aflatoxin B_1_ Adsorbent In Vivo. J. Biochem. Mol. Toxicol..

[106] Zhu L., Lei Z., Xia X., Zhang Y., Chen Y., Wang B., Li J., Li G., Yang G., Cao G. (2021). Yeast Shells Encapsulating Adjuvant AS04 as an Antigen Delivery System for a Novel Vaccine against *Toxoplasma gondii*. ACS Appl. Mater. Interfaces.

[107] Liu H., Meng Z., Wang H., Zhang S., Huang Z., Geng X., Guo R., Wu Z., Hong Z. (2021). Robust Immune Responses Elicited by a Hybrid Adjuvant Based on β-Glucan Particles from Yeast for the Hepatitis B Vaccine. ACS Appl. Bio Mater..

[108] Yang F., Meng L., Lin S., Wu F., Liu J. (2021). Polyethyleneimine-Complexed Charge-Reversed Yeast Cell Walls for the Enhanced Oral Delivery of Pseudovirus-Based Antigens. Chem. Commun..

[109] Tan C., Wang J., Sun B. (2021). Polysaccharide Dual Coating of Yeast Capsules for Stabilization of Anthocyanins. Food Chem..

[110] Lainé E., Hoffart V., Dhifallah I., Garrait G., Beyssac E. (2026). Impact of Yeast Cell Wall Incorporation on the Mucoadhesion, Stability, Oral Permeability and Release Profile of Alginate/Whey Protein Beads Loaded with Insulin. Eur. J. Pharm. Sci..

[111] Hou Y., Zhu L., Ye X., Ke Q., Zhang Q., Xie X., Piao J., Wei Y. (2024). Integrated Oral Microgel System Ameliorates Renal Fibrosis by Hitchhiking Co-Delivery and Targeted Gut Flora Modulation. J. Nanobiotechnol..

[112] Pu X., Ye N., Lin M., Chen Q., Dong L., Xu H., Luo R., Han X., Qi S., Nie W. (2021). β-1,3-d-Glucan Based Yeast Cell Wall System Loaded Emodin with Dual-Targeting Layers for Ulcerative Colitis Treatment. Carbohydr. Polym..

[113] Fan G., Cottet J., Rodriguez-Otero M.R., Wasuwanich P., Furst A.L. (2022). Metal–Phenolic Networks as Versatile Coating Materials for Biomedical Applications. ACS Appl. Bio. Mater..

[114] Chen H., Sun Y., Xu X., Ye Q. (2022). Targeted Delivery of Methotrexate by Modified Yeast β-Glucan Nanoparticles for Rheumatoid Arthritis Therapy. Carbohydr. Polym..

[115] Huang J., Zhang S., Liu D., Feng X., Wang Q., An S., Xu M., Chu L. (2024). Preparation and Characterization of Astaxanthin-Loaded Microcapsules Stabilized by Lecithin-Chitosan-Alginate Interfaces with Layer-by-Layer Assembly Method. Int. J. Biol. Macromol..

[116] Kariminia S., Shamsipur M., Mansouri K. (2024). A Novel Magnetically Guided, Oxygen Propelled CoPt/Au Nanosheet Motor in Conjugation with a Multilayer Hollow Microcapsule for Effective Drug Delivery and Light Triggered Drug Release. J. Mater. Chem. B.

[117] Musin E.V., Dubrovskii A.V., Chebykin Y.S., Kim A.L., Tikhonenko S.A. (2025). Polyelectrolyte Microcapsule-Assembled Colloidosomes: A Novel Strategy for the Encapsulation of Hydrophobic Substances. Polymers.

[118] AbouAitah K., Turk A., Bu Y., Sabbagh F., Lee M.K., Kim B.S. (2025). Nanoformulations-Based Functionalized Boron Nitride Nanosheets Delivering Nicotinamide Mononucleotide with Enhanced In Vitro Anti-Aging Properties. J. Drug Deliv. Sci. Technol..

[119] de Souza C.J.F., da Silva C.S., Ramos A.V., Garcia-Rojas E.E., Pierucci A.P.T.R. (2023). Yeast Cells-Xanthan Gum Coacervation for Hydrosoluble Bioactive Encapsulation. Int. J. Biol. Macromol..

[120] Udalova I.A., Mantovani A., Feldmann M. (2016). Macrophage Heterogeneity in the Context of Rheumatoid Arthritis. Nat. Rev. Rheumatol..

[121] Havryliuk H., Khimion L. (2019). Platelet Autologous Plasma in Post-Traumatic Knee Osteoarthritis Treatment. J. Clin. Orthop. Trauma.

[122] Haubruck P., Pinto M.M., Moradi B., Little C.B., Gentek R. (2021). Monocytes, Macrophages, and Their Potential Niches in Synovial Joints—Therapeutic Targets in Post-Traumatic Osteoarthritis?. Front. Immunol..

[123] Zhang L., Peng H., Zhang W., Li Y., Liu L., Leng T. (2020). Yeast Cell Wall Particle Mediated Nanotube-RNA Delivery System Loaded with miR365 Antagomir for Post-Traumatic Osteoarthritis Therapy via Oral Route. Theranostics.

[124] Murakami T. (2023). Atherosclerosis and Arteriosclerosis. Hypertens. Res..

[125] Libby P., Loscalzo J., Ridker P.M., Farkouh M.E., Hsue P.Y., Fuster V., Hasan A.A., Amar S. (2018). Inflammation, Immunity, and Infection in Atherothrombosis. J. Am. Coll. Cardiol..

[126] Zhang L., Li J., Kou Y., Shen L., Wang H., Wang Y., Ma R., Wu T., Yang X., Gu Y. (2024). Mechanisms and Treatment of Atherosclerosis: Focus on Macrophages. Front. Immunol..

[127] Yang K., Xiao Q., Niu M., Pan X., Zhu X. (2022). Exosomes in Atherosclerosis: Convergence on Macrophages. Int. J. Biol. Sci..

[128] Ma J., Wang Y., Xu W., Wang H., Wan Z., Guo J. (2025). Macrophage Pyroptosis in Atherosclerosis: Therapeutic Potential. Acta Biochim. Biophys. Sin..

[129] Gu X., Du L., Lin R., Ding Z., Guo Z., Wei J., Li Y. (2025). How Advanced Is Nanomedicine for Atherosclerosis?. Int. J. Nanomed..

[130] Zhang X., Xu X., Chen Y., Dou Y., Zhou X., Li L., Li C., An H., Tao H., Hu H. (2017). Bioinspired Yeast Microcapsules Loaded with Self-Assembled Nanotherapies for Targeted Treatment of Cardiovascular Disease. Mater. Today.

[131] Yin L., Peng C., Tang Y., Yuan Y., Liu J., Xiang T., Liu F., Zhou X., Li X. (2020). Biomimetic Oral Targeted Delivery of Bindarit for Immunotherapy of Atherosclerosis. Biomater. Sci..

[132] Jaecklein E., Papavinasasundaram K., Ostroff G.R., Sassetti C., Soto E.R. (2025). Targeted Delivery of Antitubercular Drugs Using Glucan Lipid Particles. Microbiol. Spectr..

